# Understanding the differences between iron and palladium in cross-coupling reactions†

**DOI:** 10.1039/c8cp07671e

**Published:** 2019-02-25

**Authors:** Xiaobo Sun, Marcus V. J. Rocha, Trevor A. Hamlin, Jordi Poater, F. Matthias Bickelhaupt

**Affiliations:** Department of Theoretical Chemistry and Amsterdam Center for Multiscale Modeling (ACMM), VU University Amsterdam De Boelelaan 1083 1081 HV Amsterdam The Netherlands f.m.bickelhaupt@vu.nl; Institute of Chemistry – Departament of Physical Chemistry, Fluminense Federal University Outeiro De São João Baptista 24020-141 Niteroi Rio de Janeiro Brazil; ICREA Pg. Lluís Companys 23 08010 Barcelona Spain; Departament de Química Inorgànica i Orgànica & IQTCUB, Universitat de Barcelona 08028 Barcelona Catalonia Spain jordi.poater@ub.edu; Institute for Molecules and Materials (IMM), Radboud University Nijmegen Heyendaalseweg 135 6525 AJ Nijmegen The Netherlands

## Abstract

We aim at developing design principles, based on quantum chemical analyses, for a novel type of iron-based catalysts that mimic the behavior of their well-known palladium analogs in the bond activation step of cross coupling reactions. To this end, we have systematically explored C–X bond activation *via* oxidative addition of CH_3_X substrates (X = H, Cl, CH_3_) to model catalysts ^m^Fe(CO)_4_^*q*^ (*q* = 0, −2; m = singlet, triplet) and, for comparison, Pd(PH_3_)_2_ and Pd(CO)_2_, using relativistic density functional theory at the ZORA-OPBE/TZ2P level. We find that the neutral singlet iron catalyst ^1^Fe(CO)_4_ activates all three C–X bonds *via* barriers that are lower than those for Pd(PH_3_)_2_ and Pd(CO)_2_. This is a direct consequence of the capability of the iron complex to engage not only in π-backdonation, but also in comparably strong σ-donation. Interestingly, whereas the palladium complexes favor C–Cl activation, ^1^Fe(CO)_4_ shows a strong preference for activating the C–H bond, with a barrier as low as 10.4 kcal mol^−1^. Our results suggest a high potential for iron to feature in palladium-type cross-coupling reactions.

## Abstract 2

We aim at developing design principles, based on quantum chemical analyses, for a novel type of iron-based catalysts that mimic the behavior of their well-known palladium analogs in the bond activation step of cross coupling reactions.

## Graphical abstract

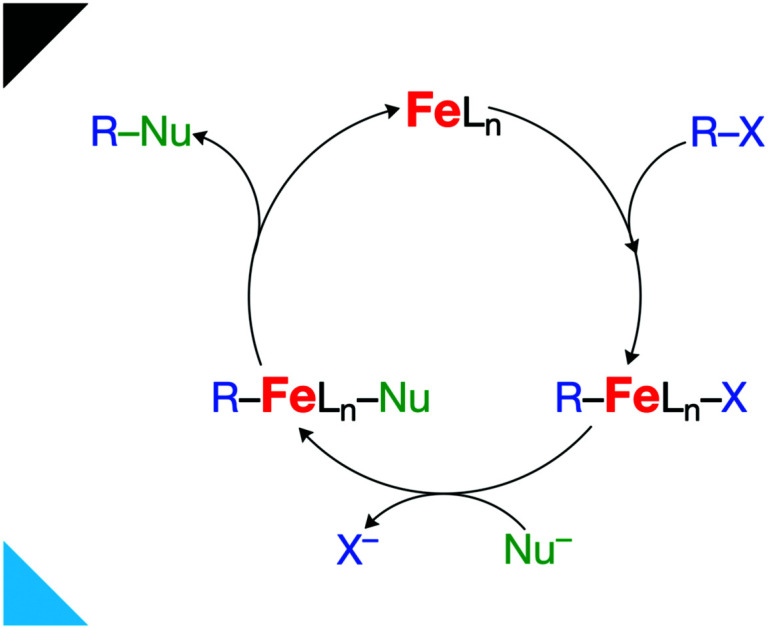

## Introduction

Catalysis is ubiquitous in modern synthetic and industrial chemistry, and plays a key role in reducing the consumption of energy and feedstocks. Yet, “designing” catalysts with the desired activity and selectivity is still a formidable task, and to a large extent, an empirical undertaking that proceeds through trial and error.^1–3^ In order to facilitate this process, a fragment-based approach, called the activation strain model^4–6^ (ASM, also known as the distortion/interaction model^7,8^) of chemical reactivity, which will be explained later on, can be used to understand how and why a certain combination of a metal center, ligands and solvent is able to selectively catalyze one particular bond in the substrate. Using this model, our group has performed a series of studies to systematically investigate the effect of a specific variation on the reactivity of the catalysts, especially for palladium in key steps for cross-coupling reactions.^9,10^ For example, we have explored not only how the reaction barrier varies when different bonds are activated by palladium,^11,12^ or different ligands are attached to palladium,^13^ but also how different metal centers perform compared to palladium.^14^

**Scheme 1:**
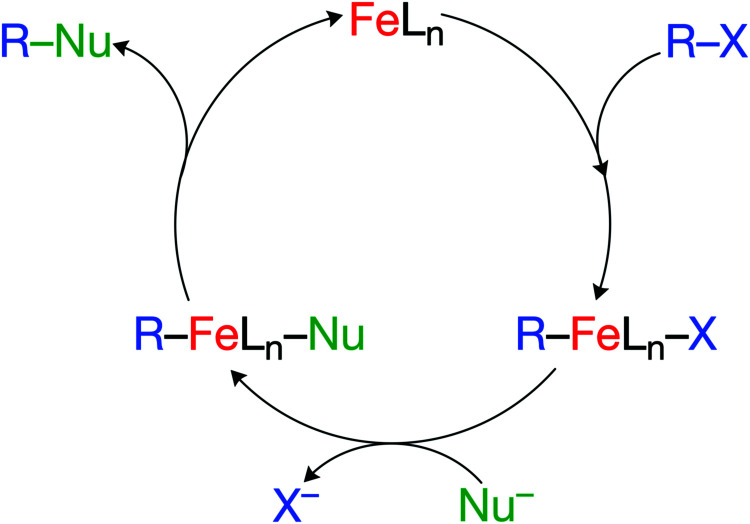
Key steps in catalytic cross-coupling model reactions.

Proceeding from the insights obtained in the above studies, we now aim at a next step: the exploration of iron's potential to take over from palladium in archetypal, closed-shell catalytic cross-coupling reactions, as illustrated by the generic catalytic cycle in Scheme 1. There are processes known in which iron-centers feature in such pathways (*vide infra*) although, in general, they react often *via* radical mechanisms.^15–17^ Our purpose, here, is not to optimize the latter. Instead, we wish to understand how and why FeL_*n*_ complexes behave in general differently from PdL_*n*_ complexes. In this way, we develop a theoretical framework that facilitates a more systematic development of iron-based cross-coupling chemistry.

Cross-coupling reactions constitute one of the most important tools for efficiently creating a bond between two carbon atoms, and have been widely investigated, both experimentally and theoretically.^18,19^ The first and generally rate-determining step in the catalytic cycle of a typical cross-coupling reaction is the activation of a bond, such as C–H, C–C and C–X (where X = halogen), by oxidative addition to a transition-metal complex. Moreover, activation of these bonds is also an important step towards the efficient conversion of abundant and inert compounds into more useful products.^15,20,21^ While iron plays an important role in other areas of modern synthetic chemistry, it has been traditionally eclipsed by other transition metals such as palladium in cross-coupling reactions.^22,23^ However, the growing concern about environmental damage and energy consumption nowadays demands cheap, nontoxic and highly selective catalysts.^24^ For this reason, iron-based catalysts, started by the pioneering work of Kochi^25–28^ in the early 1970s, and later carried on by Cahiez,^29–32^ have quickly become an important tool in the cross-coupling arsenal. Based on recent work by Fürstner,^33–37^ Nakamura and Nakamura,^38–42^ Hayashi,^43–45^ and Bedford^46–49^ new catalysts have been developed which tolerate both a rich manifold of reactivity patterns and various functional groups. However, despite the growing number of studies, their applicability in synthesis is still scarce,^3,16^ and the mechanism of iron-catalyzed cross-coupling reactions is not fully understood yet, in contrast with Pd.^50–60^

Here, we wish to explore a different idea: as opposed to developing a separate and different iron chemistry, is it possible to “teach” iron to do the tricks of palladium? This is a quantum chemical proof-of-concept study that requires several questions to be answered: what are the major electronic and structural differences between iron and palladium systems? Why, so far, have these differences made palladium the most favorite metal in cross-coupling reactions? How could iron complexes mimic the behavior of palladium catalysts by careful choice of suitable ligands? To answer these questions, we have systematically explored iron-mediated C–X bond activation *via* oxidative addition of CH_3_X substrates (X = H, Cl, CH_3_) to model catalysts ^m^Fe(CO)_4_^*q*^ (*q* = 0, −2; m = singlet, triplet) as well as selected reactions involving analogous palladium model catalysts, using relativistic density functional theory at the ZORA-OPBE/TZ2P level in combination with the activation strain model and quantitative molecular orbital (MO) theory.

There are several reasons to choose the prototypical iron-carbonyl complexes ^m^Fe(CO)_4_^*q*^ as model catalysts in our quest: (i) these model systems have the required simplicity to focus on, and uncover, the underlying physical factors of their properties and to compare them with simple bis-ligated Pd catalyst complexes; (ii) at the same time, Fe(CO)_4_ is feasible to use in the lab and has been extensively studied for its properties and interactions with various species such as hydrogen, nitrogen, hydrocarbon and so on;^61–66^ and (iii) importantly, iron-based carbonyl complexes constitute one of the most important families in organometallic chemistry, displaying a wealth of structural complexity and chemical reactivity. This stability and diversity have their roots in the very nature of the carbonyl ligand: CO has the right orbital electronic structure for balanced σ-donation through the 5σ orbital and π-backdonation through the 2π orbitals, which is crucial in the formation of stable metal–ligand bonds.^67,68^ Furthermore, CO can be easily replaced by other ligands, such as BR or PR_3_, which offer an arsenal of tuning possibilities.^69^

## Methods

### General procedure

All calculations were carried out using the Amsterdam Density Functional (ADF)^70–79^ and the quantum-regions interconnected by local descriptions (QUILD) program^80^ using relativistic density functional theory at the ZORA-OPBE/TZ2P^81–90^ level. The frozen core approximation (small frozen core) was applied in all calculations to reduce the computational cost, as the relative energies of stationary points differ by less than 1 kcal mol^−1^ if computed with or without frozen core. Our early work and extensive benchmarking^91^ (see Tables S1–S7 in the ESI†) have proven this approach to be well suited for the systems of interest. In particular, OPBE is suggested by Truhlar^92^ and Swart^93^ to accurately perform in the determination of the ground spin state of iron complexes, which is important in answering the tricky question as to whether the singlet or triplet is the ground state for Fe(CO)_4_ in our study. Geometries were optimized without any symmetry constraints. Through vibrational analysis, all energy minima and transition state structures were confirmed to be either equilibrium structures (zero imaginary frequencies) or transition states (a single imaginary frequency). The character of the normal mode associated with the imaginary frequency was analyzed to ensure that the correct transition state was found. Where computationally feasible, intrinsic reaction coordinate (IRC) calculations have been performed to obtain the potential energy surfaces (PES) of the reactions. Throughout this paper, our discussion is based on the electronic energies of the molecular systems. Notably, Gibbs free activation barriers and free reaction energies have also been calculated and the trends in reactivity remain unchanged (see Table S1 of the ESI†). The PyFrag program was used to facilitate the analyses of the potential energy surfaces (PESs).^94^ All computations, including analysis, were carried out in the gas-phase, as the trends in the oxidative insertion potential energy surfaces of iron model catalysts into H_3_C–X bonds (X = H, Cl, CH_3_) in solution (THF) remained unchanged (see Table S3, ESI†). Further details are provided in the ESI.†

### Activation strain model analysis

Insight into the overall reaction energies is obtained through activation strain model (ASM) analyses.^95^ The activation strain model of chemical reactivity is a fragment-based approach to understand the energy profile of a chemical process and to explain it in terms of the original reactants. This allows an easy assessment of the influence of geometrical deformation and the electronic structure of the catalyst and the substrate. Obviously, in the current work, our main interest is the interplay between one fragment, the catalyst, and another fragment, the substrate. Subsequently, the division of the reaction system into catalyst and substrate is used in the PyFrag computation to generate the activation strain profile. Thus, the bonding energy Δ*E* is decomposed along the intrinsic reaction coordinate (IRC) into the strain energy Δ*E*_strain_, which is associated with the geometrical deformation of the individual reactants as the process takes place, plus the actual interaction energy Δ*E*_int_ between the deformed reactants. We project the reaction coordinate on the stretch of the activated C–X bond, which has been shown to be a suitable choice.^96,97^ Furthermore, the strain energy Δ*E*_strain_ can be readily split into contributions from the deformation of the substrate and that of the catalyst (see eqn (1)).

1 Δ*E* = Δ*E*_strain_[substr] + Δ*E*_strain_[cat] + Δ*E*_int_

The interaction energy Δ*E*_int_ between the deformed reactants is further analyzed in the conceptual framework provided by the Kohn–Sham molecular orbital (KS-MO) model, using a quantitative energy decomposition scheme (see eqn (2)):^98^

2 Δ*E*_int_ = Δ*V*_elstat_ + Δ*E*_Pauli_ + Δ*E*_oi_

The term Δ*V*_elstat_ corresponds to the classical Coulomb interaction between the unperturbed charge distributions of the deformed reactants and is usually attractive. The Pauli repulsion energy Δ*E*_Pauli_ comprises the destabilizing interactions between occupied orbitals on the respective reactants and is responsible for steric repulsion. The orbital interaction energy Δ*E*_oi_ accounts for charge transfer (interaction between occupied orbitals on one fragment and unoccupied orbitals on the other fragment, including the HOMO–LUMO interactions) and polarization (empty-occupied orbital mixing on one fragment due to the presence of another fragment).

## Results and discussion

### Model catalysts

**Fig. 1:**
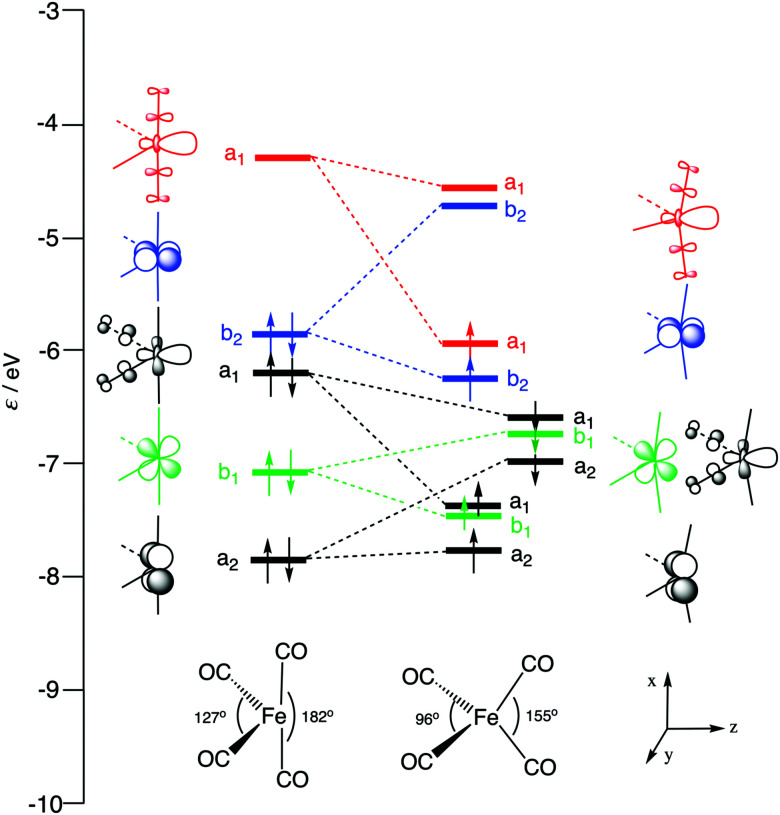
Schematic molecular orbital (MO) diagrams of ^1^Fe(CO)_4_ (left) and ^3^Fe(CO)_4_ (right: α- and β-spin levels), computed at ZORA-OPBE/TZ2P.

**Fig. 2:**
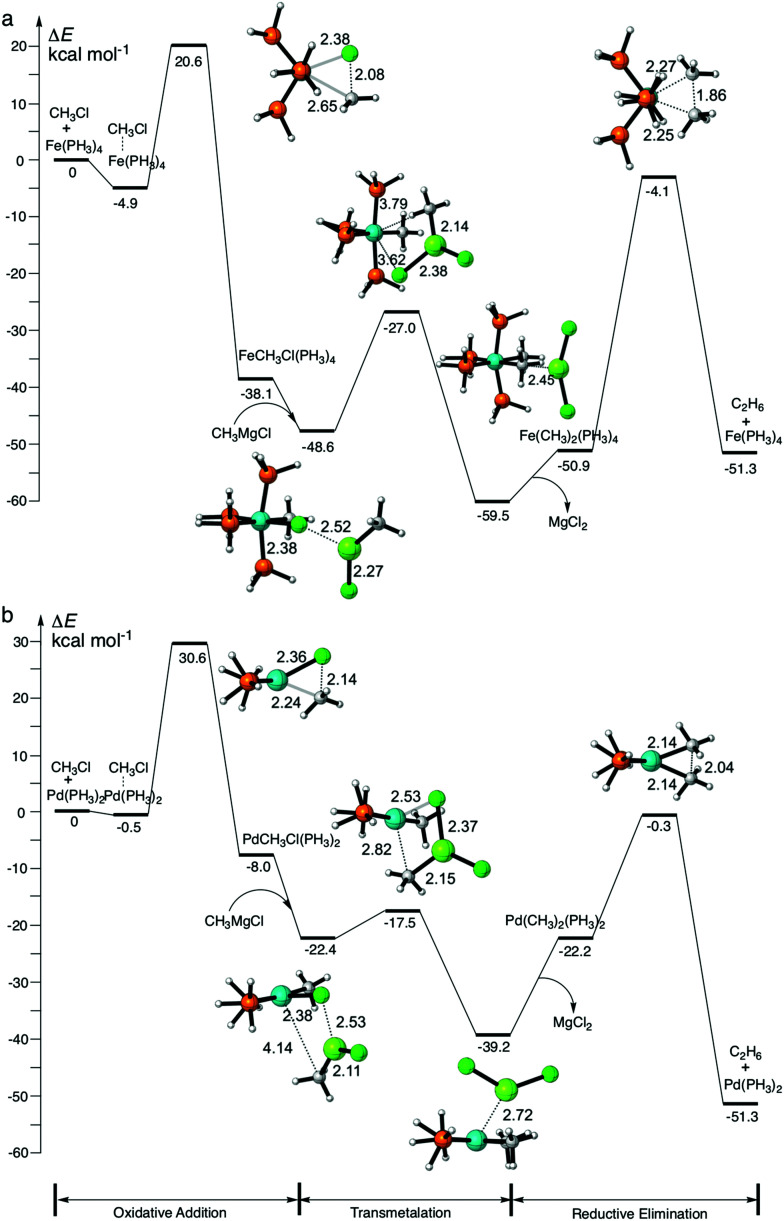
Reaction profiles and key structures (in Å) for cross coupling of chloromethane with methylmagnesium chloride, catalyzed by: (a) Fe(PH_3_)_4_ and (b) Pd(PH_3_)_2_ (see eqn (3)), computed at ZORA-OPBE/TZ2P.

**Fig. 3:**
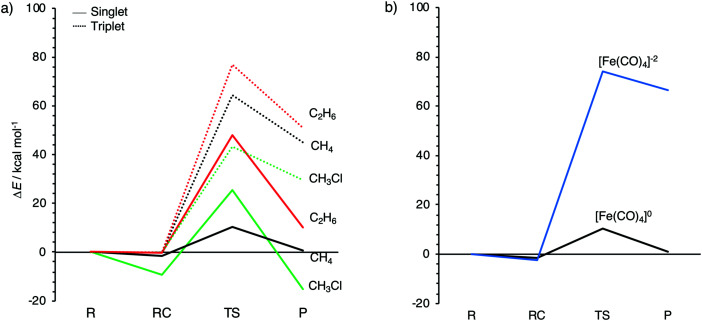
Reaction profiles for the oxidative insertion of: (a) singlet and triplet Fe(CO)_4_ into CH_3_X (X = H, Cl, CH_3_); and (b) neutral and dianionic singlet Fe(CO)_4_ into CH_4_, computed at ZORA-OPBE/TZ2P.

**Fig. 4:**
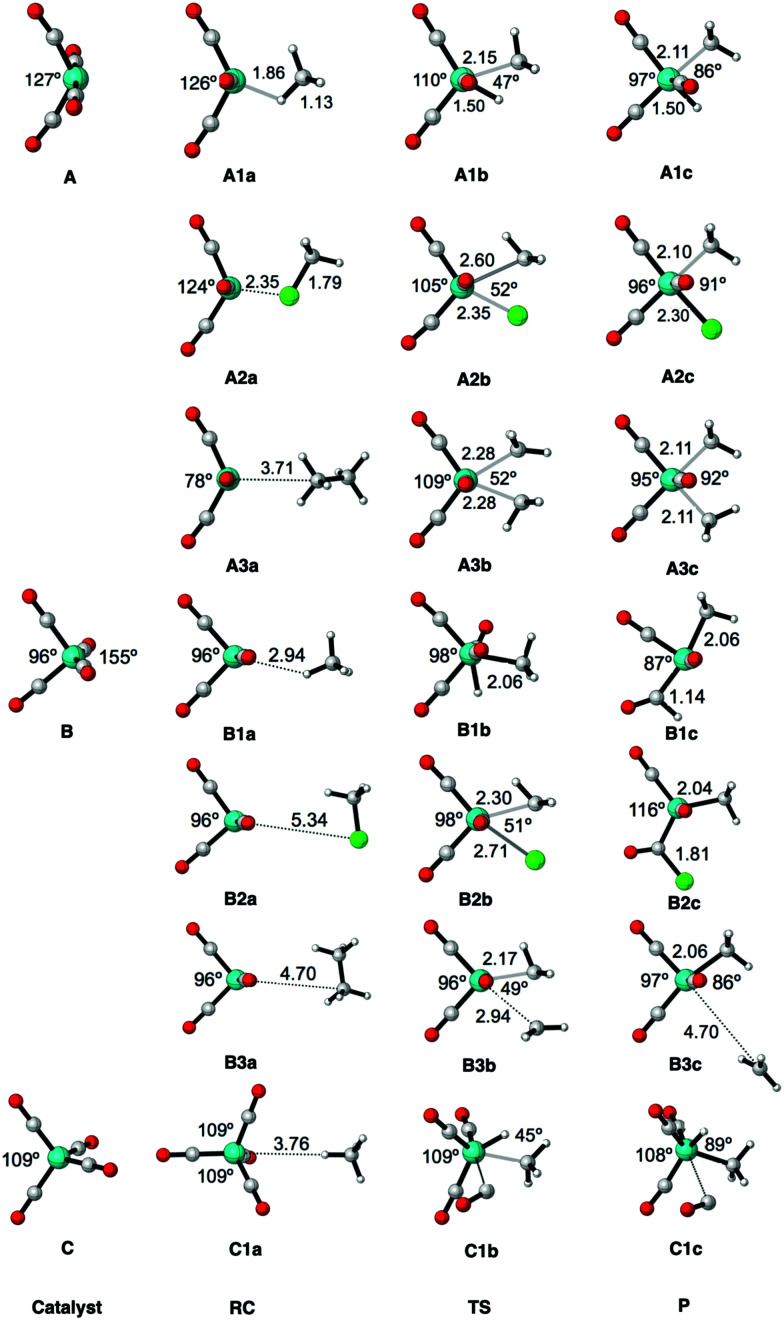
Structure parameters (the bite angle of OC–Fe–CO, and the bond lengths of Fe–X, Fe–C and C–X in Å) of the stationary points along the reaction coordinate for the oxidative insertion of Fe(CO)_4_ into CH_4_, CH_3_Cl and C_2_H_6_, computed at ZORA-OPBE/TZ2P. Uppercase letters A, B, and C represent Fe(CO)_4_ in the neutral singlet state, neutral triplet state and anionic (2−) singlet state, respectively. Numbers 1, 2, and 3 represent the three substrates CH_4_, CH_3_Cl, and C_2_H_6_, respectively. Lowercase letters a, b, and c represent the three stages along the oxidative reaction, namely the reactant complex, transition state and final product, respectively.

**Fig. 5:**
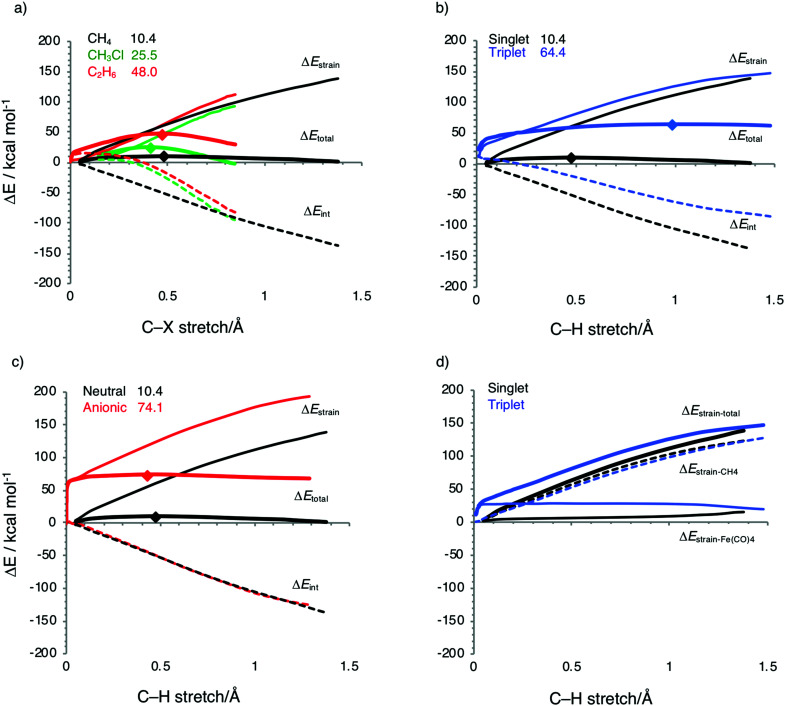
Activation strain diagrams (ASD) for the oxidative addition of: (a) CH_3_–X bonds (X = H, Cl, CH_3_: black, green, red) to singlet Fe(CO)_4_; (b) CH_3_–H bonds to singlet and triplet Fe(CO)_4_ (black and blue); and (c) CH_3_–H bonds to neutral and dianionic Fe(CO)_4_ (black and red); and (d) decomposition of the total strain energy into catalyst and substrate strain. Energy barriers relative to reactants for the TSs are also included (see upper left corner panel) and the positions of TSs are marked by dots.

**Fig. 6:**
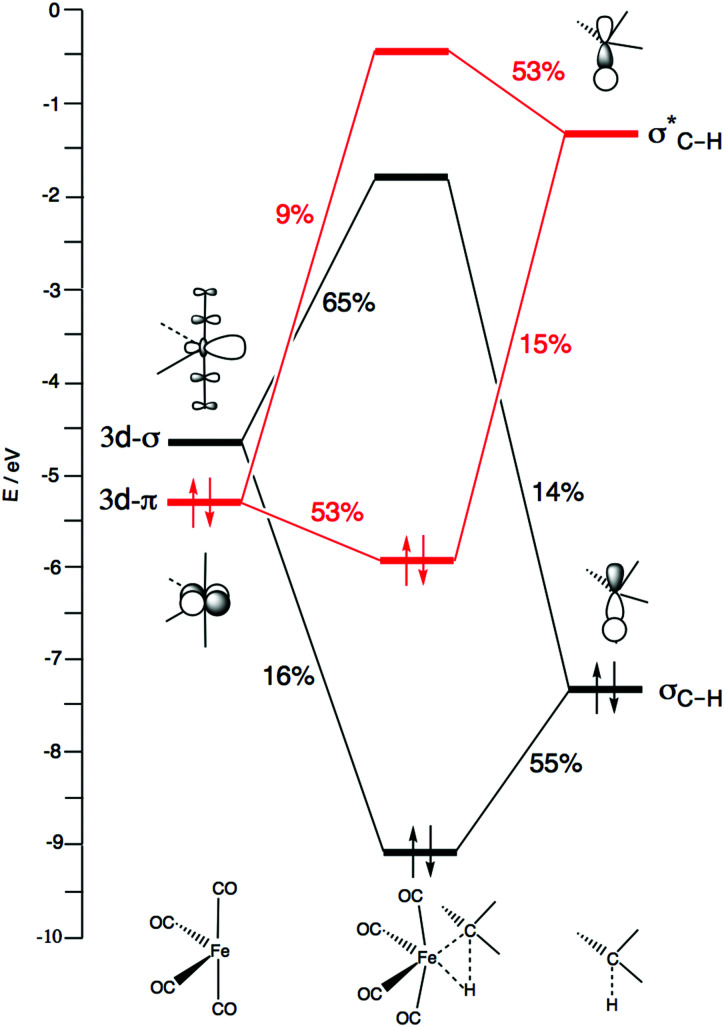
Catalyst–substrate orbital interactions in the TS for the oxidative insertion of ^1^Fe(CO)_4_ into the CH_4_ C–H bond, with gross Mulliken percentage contributions of FMOs to MOs.

**Fig. 7:**
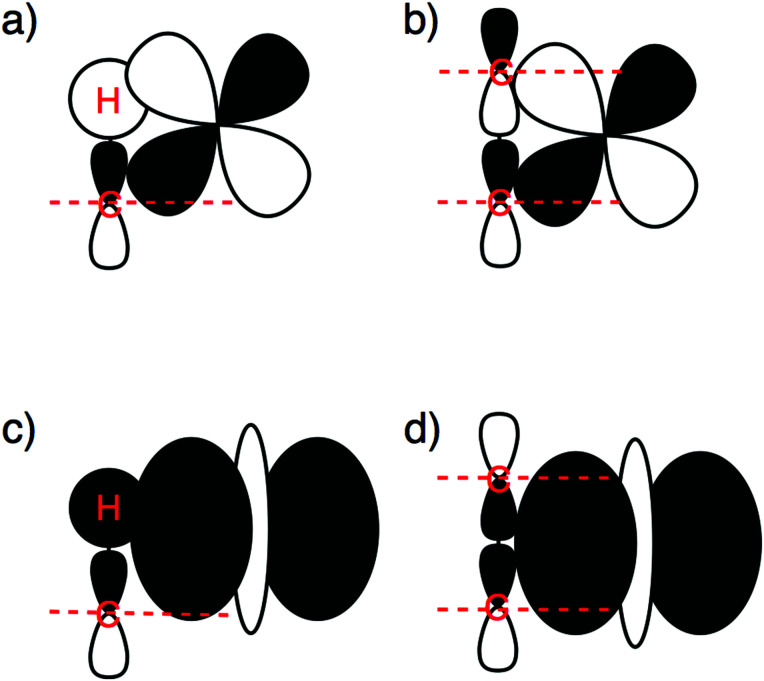
Orbital overlap pattern for catalyst–substrate π-backdonation (a and b) and σ-donation (c and d) in the case of the C–H bond (a and c) and C–Cl and C–C bonds (b and d).

**Fig. 8:**
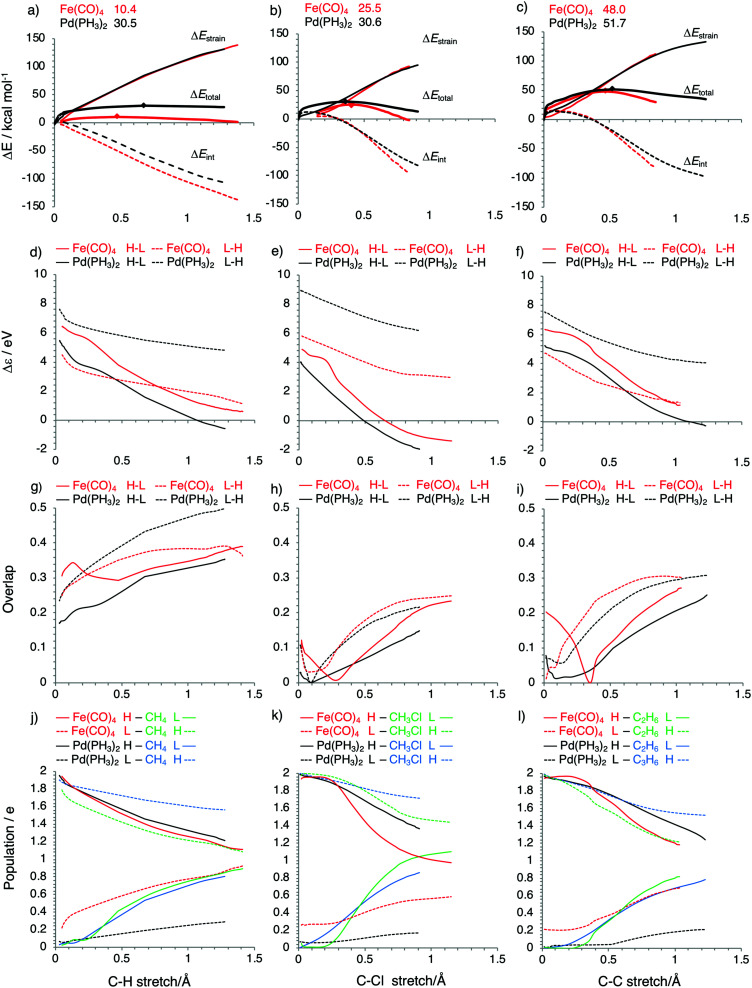
Activation strain diagrams (ASD) for the oxidative addition of Fe(CO)_4_ (red) and Pd(PH_3_)_2_ (black) into CH_3_–H (a), CH_3_–Cl (b) and CH_3_–CH_3_ bonds (c). Energy barriers relative to reactants for the TSs are also included and the positions of TSs are marked by diamonds (a–c). FMO energy gap and overlap between metal complex Fe(CO)_4_ (red) or Pd(PH_3_)_2_ (black) and CH_3_–H (d, g), CH_3_–Cl (e, h) and CH_3_–CH_3_ bonds (f, i). H–L (solid line) designates [metal-complex d_π_]–[substrate σ_c–x_*]. L–H (dashed line) designates [metal-complex d_σ_]–[substrate σ_c–x_]. (j, k and l) depict the populations of these orbitals (Fe(CO)_4_ d–π in the red solid line, Fe(CO)_4_ d–σ in the red dashed line, Pd(PH_3_)_2_ d–π in the black solid line, Pd(PH_3_)_2_ s in the black dashed line, substrate σ_c–x_ in the green or blue dashed line, substrate σ_c–x_* in the green or blue solid line).

In the following, we first examine the geometric and electronic structure of our model iron-catalysts ^m^Fe(CO)_4_^*q*^ for: *q* = 0 with m = singlet or triplet states; and for *q* = −2 with m = the singlet state. Also, we address the proof-of-concept character of this study, in particular, the fact that model iron-based catalysts of the type we focus on in this study cover all key steps of a full cross-coupling catalytic cycle, in the same way as, for example, the palladium-based model catalyst Pd(PH_3_)_2_. Subsequently, we explore the various reaction pathways for activating C–H, C–C and C–Cl bonds *via* oxidative addition to the model ^m^Fe(CO)_4_^*q*^ complexes, followed by detailed activation strain and bonding analyses that serve to uncover the physical factors that are behind the reactivity trends. In the last section, palladium-mediated reactions are included to enable the development of design principles for iron-complexes that mimic the bond-activation behavior of the palladium systems. The results of our ZORA-OPBE/TZ2P calculations are collected in Tables 1–3 and shown in Fig. 1–8 (detailed structural data are available in the ESI†).

**Table 1:** Geometry parameters (in Å and degrees) of singlet and triplet Fe(CO)_4_, and ^1^Fe(CO)_4_^2−^ a

	Fe–C_ax_	Fe–C_eq_	C–O_ax_	C–O_eq_	C_ax_–Fe–C_ax_	C_eq_–Fe–C_eq_	Fe–C_ax_–O_ax_	Fe–C_eq_–O_eq_
^1^Fe(CO)_4_	1.777	1.732	1.151	1.158	181.7	126.6	178.2	170.5
^3^Fe(CO)_4_	1.810	1.761	1.151	1.154	154.5	96.0	175.8	178.8
^1^Fe(CO)_4_^2−^	1.727	1.727	1.201	1.201	109.7	109.7	179.8	179.8

a Computed at ZORA-OPBE/TZ2P.

**Table 2:** Reaction profile (in kcal mol^−1^) for the oxidative insertion of iron and palladium model catalysts into H_3_C–X bonds (X = H, Cl, CH_3_)^a^

		RC	TS	P
^1^Fe(CO)_4_	C–H	−1.3	10.4	0.8
	C–Cl	−9.0	25.5	−15.1
	C–C	0.1	48.0	10.2
^3^Fe(CO)_4_	C–H	0.0	64.4	45.2
	C–Cl	0.0	43.3	29.4
	C–C	−0.1	76.9	51.1
^1^Fe(CO)_4_^2−^	C–H	−2.4	74.1	66.3

Pd(PH_3_)_2_	C–H	−0.2	30.5	28.1
	C–Cl	−0.5	30.6	−8.0
	C–C	−0.3	51.7	29.8
Pd(CO)_2_	C–H	−0.0	33.4	31.7
	C–Cl	−0.2	34.7	5.1
	C–C	−0.1	53.9	32.8

a Computed at ZORA-OPBE/TZ2P. ^1^Fe(CO)_4_ is 0.2 kcal mol^−1^ less stable than ^3^Fe(CO)_4_.

**Table 3:** Analysis of transition states for oxidative insertions of ^1^Fe(CO)_4_ into H_3_C–H, H_3_C–Cl and H_3_C–CH_3_ bonds^a^

	CH_4_	CH_3_Cl	C_2_H_6_
EDA (in kcal mol^−1^)			
Δ*E*_strain[“Fe”]_	6.1	5.2	4.0
Δ*E*_strain[“X”]_	54.4	31.2	57.3
Δ*E*_strain_	60.5	36.4	61.3
Δ*V*_elstat_	−104.9	−47.8	−63.6
Δ*E*_Pauli_	151.8	87.8	104.7
Δ*E*_σ_	−37.4	−22.5	−22.8
Δ*E*_π_	−49.7	−19.8	−23.4
Δ*E*_rest_	−9.9	−8.6	−8.2
Δ*E*_oi_	−97.0	−50.9	−54.4
Δ*E*_int_	−50.1	−10.9	−13.3
Δ*E*	10.4	25.5	48.0

FMO energy (in eV)			
“Fe”: d_σ_	−4.6	−4.6	−4.5
“X”: σ_c–x_	−7.3	−8.9	−7.0
Δ*ε*_donation_	2.7	4.3	2.5
“Fe”: d_π_	−5.3	−5.3	−5.3
“X”: σ_c–x_*	−1.3	−3.4	−0.9
Δ*ε*_backdonation_	4.0	1.9	4.4

FMO overlap			
〈Fe: d_σ_|X: σ_c–x_〉	0.30	0.15	0.20
〈Fe: d_π_|X: σ_c–x_*〉	0.35	0.05	0.09

FMO population (in e)			
“Fe”: d_σ_	0.42	0.37	0.25
“X”: σ_c–x_	1.44	1.73	1.62
“Fe”: d_π_	1.54	1.63	1.75
“X”: σ_c–x_*	0.58	0.36	0.35

a Computed at ZORA-OPBE/TZ2P.

Let us first inspect the three prototypical iron complexes ^1^Fe(CO)_4_, ^3^Fe(CO)_4_ and ^1^Fe(CO)_4_^2−^. Fig. 1 depicts the frontier MOs of neutral Fe(CO)_4_ and their occupation in the singlet and triplet states, *i.e.*, in ^1^Fe(CO)_4_ and ^3^Fe(CO)_4_. The triplet state is the ground state. Its open-shell nature gives rise to iron's tendency to react *via* radical pathways. But, the singlet state is only slightly, that is, 0.2 kcal mol^−1^, higher in energy than the triplet state. Herein, we are mainly interested in iron's less common closed-shell chemistry on the singlet potential energy surface (PES), which shows striking similarities and, yet, also characteristic differences to palladium chemistry.

Our computed geometries and energies of model iron complex ^1^Fe(CO)_4_^2−^, singlet ^1^Fe(CO)_4_, and triplet ^3^Fe(CO)_4_ agree well with data from previous theoretical and experimental studies.^99–105^ While the anionic d^10^-metal complex ^1^Fe(CO)_4_^2−^ is tetrahedrally coordinated,^3^ the neutral d^8^-Fe(CO)_4_ complexes, both singlet and triplet, have *C*_2v_ symmetry (see Fig. 1 and Table 1). The larger axial and equatorial OC–Fe–CO angles in ^1^Fe(CO)_4_ make this system resemble a trigonal bipyramid in which one of the equatorial sites is vacant. On the other hand, the geometry of ^3^Fe(CO)_4_ approaches more closely a distorted tetrahedron. Later on, we will see that the larger OC–Fe–CO angle of ^1^Fe(CO)_4_ is responsible for a significantly less destabilizing activation strain and, thus, lower barrier for C–X bond activation by this catalyst complex.

### Proof-of-concept character of model catalytic cycles

Next, we address the proof-of-concept character of this study. Whereas the main emphasis of our analyses is to obtain an understanding of the differences between iron- and palladium-based model catalysts in the initial, selectivity-determining step of C–X bond activation, we stress that ultimately it is, of course, not only this step, but the entire catalytic cycle that determines whether such a process is kinetically feasible. Therefore, we precede these more detailed analyses by a helicopter-view exploration of the full catalytic cycle for a representative model cross-coupling reaction for both an iron- and an analogous palladium model catalyst. For that purpose, we choose the cross coupling of chloromethane with the Grignard reagent methylmagnesium chloride leading to the formation of ethane, catalyzed by both Fe(PH_3_)_4_ and Pd(PH_3_)_2_ (eqn (3)). For our purpose, Fe(CO)_4_ is replaced by Fe(PH_3_)_4_ to allow for a more direct comparison with the model palladium system, Pd(PH_3_)_2_. Iron catalysts with carbonyl and phosphine ligands, such as Fe(CO)_4_, Fe(PH_3_)_4_, Fe(DPE)_2_, Fe(DMPE)_2_ and Fe(CO)_2_(DPPE) [DPE = H_2_PCH_2_CH_2_PH_2_, DMPE = (CH_3_)_2_PCH_2_CH_2_P(CH_3_)_2_ and DPPE = Ph_2_PCH_2_CH_2_PPh_2_] have similar yet slightly different properties and thus have been applied as models in the exploration of various reactions.^106–108^ Moreover, use of the simplified and generic model catalysts, Fe(PH_3_)_4_ and Pd(PH_3_)_2_, allows the physical factors governing their activity and selectivity in cross coupling reactions to be directly compared and understood.3
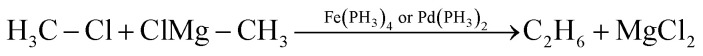


We have computationally modelled all key steps of the catalytic cycles, involving first oxidative addition, then transmetalation, and finally reductive elimination (see Scheme 1). As illustrated in Fig. 2, the overall potential energy surfaces for catalytic cycles involving Fe(PH_3_)_4_ and Pd(PH_3_)_2_ are qualitatively similar. Note that both model catalysts, Fe(PH_3_)_4_ and Pd(PH_3_)_2_, used to investigate the intrinsic properties of “real” catalysts,^9,109^ have unfavorable energetics for key steps of their respective catalyst cycles. The rate determining step for Pd(PH_3_)_2_ is the oxidative addition,^1^ while for ^1^Fe(PH_3_)_4_, it is the reductive elimination step.^2^ The insight gained in the model study is to be used, in particular, to tune these unfavorable steps in the model processes to achieve the desired (lower) barriers while keeping also the overall energy-span of the mechanism small.^110^

A rather unexpected finding is that Fe(PH_3_)_4_ has a 10.0 kcal mol^−1^ lower barrier than Pd(PH_3_)_2_ in the oxidative-addition step. This points to the enhanced bond activating capability for iron-based catalysts compared to the ubiquitous palladium analogs. Other than the previously mentioned differences, similar qualitative features emerge when comparing the catalytic cycles for Fe(PH_3_)_4_ and Pd(PH_3_)_2_. Thus, the use of our generic catalysts is justified and is a useful tool to understand the fundamental differences between iron- and palladium-based model catalysts.

### General reaction profiles

The aim of the present work is to design iron-based catalysts that, as closely as possible, mimic the mechanism of bond activation for cross-coupling as performed by palladium catalysts. With this in mind, we have chosen three prototypical C–X bond activations that feature in palladium-catalyzed cross-coupling mechanisms, namely, that of the methane C–H, the chloromethane C–Cl, and the ethane C–C bond, and explored their activation by the three iron carbonyl complexes introduced above. The computed energies, relative to the reactants, of the stationary points along all seven model reactions are collected in Table 2. The corresponding energy profiles are depicted in Fig. 3. In addition, geometries and designations of the stationary points located for each model reaction are depicted in Fig. 4.

The oxidative insertions of the iron complexes start from a weakly bound reactant complex (RC), at only 0 to −9.0 kcal mol^−1^ relative to the separate reactants, in which CH_3_–X (X = H, Cl, CH_3_) coordinates *via* its C–X bond (or *via* its C–X and a C–H bond) to the iron center (see Table 2 and Fig. 4). The only RC of a more substantial stability is that of the chloromethane C–Cl bond coordinating to the singlet ^1^Fe(CO)_4_ (**A2a**), at −9.0 kcal mol^−1^. The reactant complexes of neutral Fe(CO)_4_ have a singlet ground state in the case of C–H and C–Cl and a triplet ground state for C–C activation; the latter is, however, only 0.2 kcal mol^−1^ above the singlet state. Proceeding from the RC, the catalyst approaches the C–X bond, which elongates as we approach the transition state (TS), which is a triangular [FeCX] unit involving partially formed Fe–C and Fe–X bonds and a partially broken C–X bond. The lowest barriers in all cases occur for the neutral singlet-complex ^1^Fe(CO)_4_ (see Fig. 3 and Table 2). In the case of the anionic d^10^-iron catalyst, the occurrence of a 20-electron species in the reaction with methane is prevented as one CO ligand begins to dissociate. This ligand dissociation also generates extra room for the approaching C–H bond. Our activation strain analyses reveal that this metal–ligand bond breaking causes a larger strain energy in ^1^Fe(CO)_4_^2−^ and, therefore, a substantially higher energy barrier than in the reactions of the neutral d^8^-iron complex ^1^Fe(CO)_4_: 74.1 *versus* 10.4 kcal mol^−1^ (see Fig. 3b). The TS geometries for ^1^Fe(CO)_4_ and ^3^Fe(CO)_4_ are quite similar (see Fig. 4). Nevertheless, the former has significantly lower barriers than the latter: 10.4 *vs.* 64.4 kcal mol^−1^ for C–H, 25.5 *vs.* 43.3 kcal mol^−1^ for C–Cl, and 48.0 *vs.* 76.9 kcal mol^−1^ for C–C bond activation, respectively. Our activation strain analyses reveal that this increase in barrier height, from ^1^Fe(CO)_4_ to ^3^Fe(CO)_4_, is associated with a more destabilizing catalyst strain associated with the necessity of a larger widening of the initially smaller OC–Fe–CO angle in the more tetrahedral ^3^Fe(CO)_4_ complex (*vide infra*).

The reactions involving the neutral singlet complex ^1^Fe(CO)_4_ proceed not only with the lowest barrier, but also lead to the most stable products (P): in all cases, this is the direct-insertion product, in which the C–X bond has been effectively reduced and broken (see P, in Fig. 3). The reaction energies of ^1^Fe(CO)_4_ are 0.8, −15.1, and 10.2 kcal mol^−1^ for C–H, C–Cl, and C–C activation (see Table 2 and Fig. 3). This has to be compared with the substantially more endothermic reaction energies of the triplet complex ^3^Fe(CO)_4_, which are 45.2, 29.4 and 51.1 kcal mol^−1^, respectively. These observations agree with the experimental findings by Poliakoff and Turner, who suggested that the adduct (CO)_4_–HFeCH_3_ has a singlet ground state.^99,100^ The associated radical reactions differ in nature from those of the closed-shell reactions of the singlet model catalyst. These radical reactions comprise the transfer of one methyl group of the substrate to iron under formation of an electron-pair bond while the X-group either leaves (in the case of CH_3_–CH_3_) or migrates to the carbon of a CO ligand (in the case of CH_3_–H and CH_3_–Cl). The most endothermic insertion among our model reactions is C–H activation by the anionic d^10^-complex ^1^Fe(CO)_4_^2−^ with a reaction energy of 66.3 kcal mol^−1^. Note that the straight oxidative-insertion product is not stable for both ^3^Fe(CO)_4_ and ^1^Fe(CO)_4_^2−^: in the former case, an X˙ radical migrates to the carbon of a CO ligand (C–H and C–C) or it dissociates and leaves (C–C). In the latter case, that is, for ^1^Fe(CO)_4_^2−^ + CH_4_, a CO ligand departs (see Fig. 4). In this way, the formation of an unfavorable 20-electron species is avoided.

In addition to oxidative addition, we have also considered the S_N_2 reaction path for the activation of CH_3_X substrates (X = H, Cl, CH_3_) by the anionic iron complex ^1^Fe(CO)_4_^2−^. It has been suggested that Na_2_Fe(CO)_4_ (Collman's reagent) proceeds through a rapid S_N_2 reaction with alkyl bromides.^111^ Our calculations indeed find a low barrier for C–Cl activation, but high barriers for C–H and C–C activation. As we wish to focus on developing design principles for tuning the oxidation addition step of model iron catalysts, we provide the S_N_2 results in the ESI.†

In conclusion, the kinetically and thermodynamically most favorable pathways proceed *via* oxidative addition on the singlet-state energy surfaces. The associated triplet–singlet interconversion of the initial iron-carbonyl complex is a common phenomenon for first row transition-metals, especially for those that exhibit multiple spin states, such as iron (*cf.* spin-cross reaction).^112,113^

### Activation strain analysis

Next, we analyze the origin of the trends in reactivity and selectivity for the oxidative addition of iron-based catalyst complex Fe(CO)_4_ to the series of archetypal C–X bonds (X = H, Cl, CH_3_) by means of the activation strain model and Kohn–Sham molecular orbital analyses. A comparison with the corresponding palladium-mediated reactions follows, later on. Our activation strain analyses show that the reaction barrier increases from C–H to C–Cl activation because of a weaker catalyst–substrate interaction, whereas the increase from C–Cl to C–C activation arises from an increase in the destabilizing activation strain. The physical factors behind the variation in strain and interaction curves for the three types of bonds are similar to those behind the corresponding trends in reactivity of palladium-mediated C–X bond activation, as will be explained later on.^11^

Before we address the catalytic activity of ^1^Fe(CO)_4_, we first examine why ^3^Fe(CO)_4_ and ^1^Fe(CO)_4_^2−^ are not active and thus are not viable candidates for designing catalysts for cross coupling reactions. The substantial rise in barriers from ^1^Fe(CO)_4_ to either ^3^Fe(CO)_4_ or ^1^Fe(CO)_4_^2−^ mainly originates from a more destabilizing strain energy. For example, the strain energy is almost 60 kcal mol^−1^ higher for ^1^Fe(CO)_4_^2−^ than for ^1^Fe(CO)_4_ in the early stage of addition to the C–H bond (Fig. 5c), which pushes up the total energy immediately. The comparison of Fig. 5b and d shows that this prohibitively high strain mainly comes from the iron complex, which occurs because the ^3^Fe(CO)_4_ and ^1^Fe(CO)_4_^2−^ complexes are (near-)tetrahedral and lack an open site for docking the incoming substrate, at variance to ^1^Fe(CO)_4_, which has an open equatorial position in its incomplete trigonal bipyramidal geometry (Fig. 4). As a consequence, essentially no deformation is needed in the case of ^1^Fe(CO)_4_ to coordinate the incoming substrate, while ^3^Fe(CO)_4_ and ^1^Fe(CO)_4_^2−^ must undergo a substantial structural deformation. The axial CO–Fe–CO angle for ^3^Fe(CO)_4_ is widened from 155° in equilibrium geometries to 165°, 170° and 168° in TS for C–H, C–Cl and C–C activation, respectively. The larger expansion of the smaller angle (109°) eventually causes the dissociation of one ligand from the metal center in the case of ^1^Fe(CO)_4_^2−^. Note that the bending of the bite angle to make room for the approaching substrate is crucial, to avoid otherwise even stronger steric repulsion between the catalyst and the substrate.^13,114^

Next, we continue with our analyses of the physical factors behind the selectivity of ^1^Fe(CO)_4_ towards C–H, C–Cl and C–C activation, followed by the core of our work, that is, a comparison of the underlying factors of the similarities and differences in activity between our iron model catalyst and archetypal d^10^-PdL_2_ model catalysts.

### C–H, C–Cl and C–C bond activation by ^1^Fe(CO)_4_

As pointed out above, the barrier for ^1^Fe(CO)_4_-mediated bond activation increases from 10.4 to 25.5 to 48.0 kcal mol^−1^ along C–H, C–Cl and C–C bonds (see Table 2). The increase in the barrier from C–H to C–Cl and C–C activation is caused by two factors. One factor is the delay in the interaction curves Δ*E*_int_ for the two latter bonds (see Fig. 5a). In the case of C–H activation, the interaction curve Δ*E*_int_ becomes steadily more stabilizing, right from the beginning of the reaction. This makes the barrier lowest for C–H activation despite a relatively unfavorable strain curve which is comparable to that of C–C activation. At variance, in the case of C–Cl and C–C activation, the build-up in interaction energy Δ*E*_int_ lags behind until the C–X bond is stretched sufficiently. Only then do the Δ*E*_int_ curves for C–Cl and C–C gain quickly and eventually catch up with the Δ*E*_int_ curve for C–H activation (see Fig. 5a). We come back to the different behavior in the interaction curves in a moment.

The second factor behind the trend in C–X bond activation is the higher strain curve for C–C compared to C–Cl activation, which causes the barrier for C–C activation to be highest. The elongation and eventual breaking of the covalent C–X bond is the main source of the strain energy, while the bending of the OC–Fe–CO angle contributes only a little (see Fig. 4 and 5d). The more destabilizing strain from C–Cl to C–C activation simply reflects the higher bond strength of the latter bond (see also ref. 11).

To understand why C–Cl and C–C bond activations have a delayed interaction, we further analyzed the bonding mechanism behind the interaction energy profiles by means of Kohn–Sham molecular orbital analysis. All three C–X bond activations have in common that the basic interaction pattern consists mainly of two features: (i) backdonation from the d_π_ of Fe(CO)_4_ to the σ_C–X_* of CH_3_X; and (ii) donation from the σ_C–X_ of CH_3_X to the d_σ_ of Fe(CO)_4_. This bonding mechanism is depicted in Fig. 6 for the case of oxidative addition of CH_4_ to ^1^Fe(CO)_4_. The remaining three d-type orbitals of Fe are not included since they are involved only very weakly in the bonding, or not at all. Our catalyst–substrate bonding analyses at the transition states reveal that the stabilizing electrostatic (Δ*V*_elstat_) and orbital-interaction (Δ*E*_oi_) contributions to the interaction energy Δ*E*_int_ are of comparable magnitude and, at this stage of the reaction, outweigh the Pauli repulsion between occupied orbitals in the activation of all three bonds, C–H, C–Cl and C–C (see Table 3). Note also that the orbital overlaps, the extent of charge transfer from the HOMO to the LUMO in both π-backdonation and σ-donation, and the strength of orbital interactions Δ*E*_oi_ are substantially larger for C–H than for C–Cl and C–C activation.

Now, there is a fundamental difference between the σ_C–H_ and σ* orbitals of the C–H bond and the σ_C–X_ and σ_C–X_* orbitals of the C–Cl and C–C bonds, which is responsible for the delay in the catalyst–substrate interaction in the case of the two latter bonds. Fig. 7 schematically depicts the HOMO and LUMO of our three substrates CH_3_X, *i.e.*, σ_C–X_ and σ_C–X_*, which are composed of the in-phase and out-phase combination of orbitals between CH_3_ and the X unit (p-type for CH_3_ and Cl, 1s for H). Therefore, the σ_C–X_ and σ_C–X_* orbitals of the C–Cl and C–C bonds have one additional nodal surface on the X moiety as compared to the σ_C–H_ and σ_C–H_* orbitals of the C–H bond. This is depicted in Fig. 7, which also shows how the additional nodal surface of the C–Cl and C–C orbitals cuts into the lobes of the metal d orbital, in the early stages of C–Cl and C–C activation reactions at which the bonds have not yet been elongated much. This results in poor catalyst–substrate orbital overlap and thus less stabilizing π-backdonation and σ-donation for C–Cl and C–C activation compared to C–H activation, as shown by our EDA-NOCV^115,116^ calculations. Accordingly, the Δ*E*_π_ and Δ*E*_σ_ terms, which quantity the backdonation from the d_π_ of Fe(CO)_4_ to the σ_C–X_* of CH_3_X and the donation from the σ_C–X_ of CH_3_X to the d_σ_ of Fe(CO)_4_, respectively, are more stabilizing in the TS for C–H activation (Δ*E*_π_ = −49.7 and Δ*E*_σ_ = −37.4 kcal mol^−1^) than in the TS for C–Cl (Δ*E*_π_ = −19.8 and Δ*E*_σ_ = −22.5 kcal mol^−1^) and C–C activation (Δ*E*_π_ = −23.4 and Δ*E*_σ_ = −22.8 kcal mol^−1^; Table 3). Also, note that the net interaction energy curve Δ*E*_int_ for C–Cl and C–C activation is even slightly positive, that is, repulsive, in the early stages of C–Cl and C–C activation, because the weak orbital interactions cannot overcome the unfavorable Pauli repulsion between occupied orbitals (see Fig. 5a).

Only in more advanced stages of the reaction, that is, after the C–Cl and C–C bonds have been stretched sufficiently to move the p-nodal surfaces of the substrate σ_C–X_ and σ_C–X_* orbitals out of the way of the iron 3d_σ_ and 3d_π_ lobes, do favorable overlap and interaction energy Δ*E*_int_ build up, and quickly catch up with the overlap and interaction values in the case of C–H activation (see Fig. 5 and also the discussion later on). Note that the steeper interaction curves Δ*E*_int_ in these more advanced stages of the reaction pull the TS for C–Cl and C–C activation to an earlier point along the reaction coordinate than the TS for C–H activation. Interestingly, this constitutes an example of anti-Hammond behavior: the more endothermic C–C activation reaction has a more reactant-like, not a more product-like, TS than C–H activation (see Fig. 4 and especially 5).

### Iron *versus* palladium complexes

Finally, we turn to our initial question: is it possible to “teach” iron to do the tricks of palladium? The answer is a clear yes! We have seen this already in the discussion above: ^1^Fe(CO)_4_ can activate C–X bonds to an extent similar to d^10^-PdL_2_ complexes. Here, we undertake a more detailed and direct comparison of ^1^Fe(CO)_4_- *versus* Pd(PH_3_)_2_- and Pd(CO)_2_-mediated activation of methane C–H, chloromethane C–Cl, and ethane C–C bonds *via* oxidative addition. We choose these two palladium-d^10^ model catalysts because Pd(PH_3_)_2_ is, electronically, a representative model for the often bulkier palladium–phosphine complexes used in practice,^109^ whereas Pd(CO)_2_ allows for a more systematic comparison with the iron-carbonyl complex Fe(CO)_4_. Note that we recomputed the selected palladium-mediated bond activation pathways with the DFT approach of the present study, ZORA-OPBE/TZ2P, for a consistent comparison (see Table 2). Earlier work^13,117^ on the activity of Pd(PH_3_)_2_ and Pd(CO)_2_ was done with ZORA-BLYP/TZ2P, which yields the same reactivity trends, however slightly, *i.e.*, by up to 1.8 kcal mol^−1^, different barriers or reaction energies.

We recall the main observations. In the first place, our iron-based model catalyst ^1^Fe(CO)_4_ is similarly, in fact, even slightly more reactive towards C–X bond activation than the archetypal palladium-based complexes Pd(PH_3_)_2_ and Pd(CO)_2_ (see Table 2). Furthermore, the iron model catalyst ^1^Fe(CO)_4_ achieves a particularly low barrier for C–H activation, even lower than for C–Cl activation. This contrasts with the palladium complexes Pd(PH_3_)_2_ and Pd(CO)_2_ which both activate C–H and C–Cl bonds *via* nearly identical reaction barriers (see Table 2). This is in line with an early experimental observation that showed the d^8^-iron complex Fe(DMPE)_2_ [DMPE = 1,2-bis(dimethylphosphino)ethane], which is similar in geometry to ^1^Fe(CO)_4_, to undergo oxidative addition to unactivated alkane C–H bonds at temperatures below −90 °C.^108^ In contrast, palladium-based cross coupling reactions tend to activate carbon–halogen bonds, not carbon–hydrogen bonds.^1,18,19^ This indicates that properly designed iron-catalysts can be used not only to replace palladium analogs, but they may also be deployed to achieve a different selectivity.

The results of our analyses of ^1^Fe(CO)_4_- and Pd(PH_3_)_2_-induced activation of C–H, C–Cl and C–C bonds are collected and one-on-one compared in Fig. 8, which depicts the activation strain diagrams (ASD), FMO energy gaps, and FMO overlaps as well as the FMO populations for all reactions: red and black curves in each subdiagram for ^1^Fe(CO)_4_*versus* Pd(PH_3_)_2_ reactions, respectively. First, we focus on the activation of the C–H bond, for which the largest difference in barriers occurs: 30.5 *vs.* 10.4 kcal mol^−1^ for Pd(PH_3_)_2_ and Fe(CO)_4_, respectively. The corresponding ASD in Fig. 8a shows a clearly more stabilizing interaction curve Δ*E*_int_ for Fe(CO)_4_ along the entire reaction coordinate, leading to an earlier and lower barrier than for Pd(PH_3_)_2_. The strain curves Δ*E*_strain_ essentially coincide because the main source of strain energy is in both cases the breaking of the C–H bond.

The more stabilizing interaction energy curve for Fe(CO)_4_ comes from the characteristic difference that Fe(CO)_4_ has an incomplete d-shell, which features both a high-energy dπ HOMO and a low-energy dσ LUMO that can participate in strong π-backdonation as well as strong σ-donation, respectively (see Fig. 6, *vide supra*). The Pd(PH_3_)_2_ complex has a filled d-shell, which still provides a high-energy HOMO that can be deployed only for strong π-backdonation. The LUMO of Pd(PH_3_)_2_ is a higher-energy Pd-5s derived orbital, which is less capable of entering into a favorable σ-donation interaction. Thus, as can be seen in Fig. 8d–f, the LUMO–HOMO energy gap involving σ-donation is significantly larger for Pd(PH_3_)_2_ (black dashed curves) than for Fe(CO)_4_ (red dashed curves). Pd(PH_3_)_2_ thus has weak σ-donation, especially in the reaction with CH_4_. This is also reflected by the hardly changing populations of the Pd(PH_3_)_2_ 5s-type LUMO and the CH_4_ σ_C–H_ HOMO, which remain relatively close to 2 and 0, respectively (see Fig. 8j). For comparison, π-backdonation in the case of Pd(PH_3_)_2_ is as strong as that for Fe(CO)_4_, as reflected by the small orbital-energy gap, the favorably large overlap and the relatively strong charge transfer from d_π_ to σ_C–H_* (see Fig. 8d, g and j).

However, the σ-donation of ^1^Fe(CO)_4_ does not immediately come into action in the cases of C–Cl and C–C bond activations. This is why the energy barrier is reduced only slightly from Pd(PH_3_)_2_ to Fe(CO)_4_: from 30.6 to 25.5 kcal mol^−1^ for C–Cl and from 51.7 to 48.0 kcal mol^−1^ for C–C (see Table 2 and Fig. 8). This is so even though for the ^1^Fe(CO)_4_-induced bond activation the orbital-energy gap for σ-donation is still the smallest and charge transfer the strongest (see Fig. 8). However, for the C–Cl and C–C bond activation, the overlap interferes with the orbital-energy gap and codetermines the reactivity trend. Similar to the situation for the iron complex, the overlap is considerably smaller for C–Cl and C–C than for C–H activation by Pd(PH_3_)_2_. This again originates from the delayed donation and backdonation interaction, and is again caused by the cancelation between the d orbital of the metal complex and the substrate frontier orbitals at the early stage of the reaction (see Fig. 7a and b). As shown in Fig. 8h and i, due to the cancelation, the overlap for both the iron and the palladium complex remains close to zero at first and then increases quickly once the C–X bond is stretched enough. The small overlaps at early stages of the reaction lead to weak orbital interactions and, consequently, to higher energy barriers. Even though the poor overlap situation leaves little room for σ-donation to play a role, a close look at Fig. 8b and c shows that stronger orbital interaction for Fe(CO)_4_, due to the smaller orbital-energy gap, is still noticeable. Therefore, the Δ*E*_int_ curves decrease more steeply for Fe(CO)_4_ than for Pd(PH_3_)_2_ at the later stages of the reactions.

The above comparison reveals an important difference in the interaction pattern between Pd and Fe complexes and substrates. Because of the saturated d^10^-shell and high-energy s orbital of Pd complexes, π-backdonation is usually the determining interaction and the focus of tuning. The employment of ligands that can push-up the palladium d orbitals, such as σ-donating ligands, can reduce the orbital-energy gap and enhance the catalyzing capability. The approach however differs if one wishes to tune iron-d^8^ catalysts: here, one has to consider both π-backdonation to and σ-donation from the substrate. How to achieve the simultaneous tuning of these two orbital mechanisms in the design of iron-complexes for C–X bond activation and catalytic cross coupling is the subject of future work in our laboratory.

## Conclusions

Closed-shell iron-d^8^ complexes can be excellent candidates for replacing the more classical palladium-d^10^ systems in catalytic cross-coupling reactions. Our proof-of-concept quantum chemical investigation shows that a simple model system such as Fe(CO)_4_ has access to viable, non-radical pathways for C–X bond activation that closely mimic the oxidative-addition pathways of PdL_2_ complexes. This follows from our detailed analyses of the similarities of, and differences between, the reactivity of singlet ^1^Fe(CO)_4_, triplet ^3^Fe(CO)_4_, Fe(CO)_4_^2−^, Pd(CO)_2_ and Pd(PH_3_)_2_ towards C–H, C–Cl, and C–C bonds, using the activation-strain model in combination with relativistic density functional theory. The full catalytic cycles associated with the cross coupling of chloromethane with the Grignard reagent methylmagnesium chloride catalyzed by our model iron-based catalyst, Fe(PH_3_)_4_, and a prototypical palladium catalyst, Pd(PH_3_)_2_, exhibit similar qualitative features, thus justifying the use of the generic Fe(L)_4_ catalyst for our current investigations. Interestingly, the oxidative addition is the rate determining step for Pd(PH_3_)_2_, while the reductive elimination step plays a more important role for Fe(PH_3_)_4_.

In fact, Fe(CO)_4_ is even slightly more active in closed-shell (*i.e.*, singlet-state) activation of representative C–X bonds than archetypal PdL_2_ complexes. There are two major reasons for the good performance of ^1^Fe(CO)_4_. One is that FeL_4_ complexes, such as singlet ^1^Fe(CO)_4_, have an incomplete valence d^8^ shell. Consequently, they do not only have a high-energy d_π_ HOMO for effective π-backdonation to the σ_C–X_* LUMO of the substrate, a feature they share with the d^10^-complexes of palladium, but also possess an empty 3d_σ_ orbital, which is at relatively low energy as compared to the empty 5s-derived LUMO of palladium systems. This low-energy d_σ_ LUMO of the iron complex can enter into a stronger, more stabilizing orbital interaction with the occupied σ_C–X_ orbital of the substrate resulting in a lower reaction barrier. A second reason for the good performance of ^1^Fe(CO)_4_ is that ^1^FeL_4_ complexes adopt the geometry of a “trigonal bipyramid missing one equatorial ligand”. This gap in the coordination sphere can straightforwardly accommodate the incoming substrate without inducing much activation strain in the catalyst complex during the bond activation process.

Interestingly, ^1^Fe(CO)_4_ favors C–H over C–Cl activation, at variance to archetypal PdL_2_ complexes, which show more facile C–Cl than C–H activation. The reason is that the additional σ-donation into the 3d_σ_ LUMO, taking place in the iron-mediated reactions, achieves the most beneficial orbital overlap with the σ_C–H_ orbital, which is essentially 2p_σ_(C) + 1s(H). Both C–Cl and C–C activation suffer from the cancelation of orbital overlap with the iron 3d_σ_ that arises from the additional 2p-nodal surface in their σ_C–X_ orbitals, which are essentially 2p_σ_(C) + *n*p_σ_(X). For all model catalysts, C–C activation has the highest barrier because this is the strongest bond in the series, giving rise to the highest activation strain.

## Conflicts of interest

There are no conflicts to declare.

## Supplementary Material

CP-021-C8CP07671E-s001
