# Supplementary material for: Understanding the differences between iron and palladium in cross-coupling reactions
Source: Phys Chem Chem Phys. 2019 Feb 25;21(19):9651–64. doi: 10.1039/c8cp07671e (PMC8610147; doi:10.1039/c8cp07671e)
Supplement: CP-021-C8CP07671E-s001 [file CP-021-C8CP07671E-s001.pdf]

Electronic Supplementary Material (ESI) for Physical Chemistry Chemical Physics.
This journal is © the Owner Societies 2019

# Understanding the Differences Between Iron and Palladium in Cross-Coupling Reactions

Xiaobo Sun,[a] Marcus V. J. Rocha,[ab] Trevor A. Hamlin,[a] Jordi Poater,*[c]
and F. Matthias Bickelhaupt*[ad]

[a] *Department of Theoretical Chemistry and Amsterdam Center for Multiscale Modeling (ACMM), VU University Amsterdam, De Boelelaan 1083, 1081 HV Amsterdam, The Netherlands. E-mail: f.m.bickelhaupt@vu.nl*
[b] *Institute of Chemistry – Departament of Physical Chemistry, Fluminense Federal University, Outeiro De São João Baptista, 24020-141 Niteroi, Rio de Janeiro, Brazil.*
[c] *ICREA, Pg. Lluís Companys 23, 08010 Barcelona, Spain and Departament de Química Inorgànica i Orgànica & IQTCUB, Universitat de Barcelona, 08028 Barcelona, Catalonia, Spain. E-mail: jordi.poater@ub.edu*
[d] *Institute for Molecules and Materials (IMM), Radboud University Nijmegen, Heyendaalseweg 135, 6525 AJ Nijmegen, The Netherlands.*

## Contents

**Table S1.** Electronic and, in parentheses, Gibbs free energies relative to reactants for the oxidative insertion of iron model catalysts into $H_3C–X$ bonds (X = H, Cl, $CH_3$), computed at ZORA-OPBE/TZ2P.

**Table S2.** Electronic energy with frozen core (small) and, in parentheses, without frozen core (in kcal $mol^{-1}$) for the oxidative insertion of iron model catalysts into $H_3C–X$ bonds (X = H, Cl, $CH_3$), computed at ZORA-OPBE/TZ2P.

**Table S3.** Electronic energy in gas phase (in kcal $mol^{-1}$) and, in parentheses, in solution (THF) relative to reactants for the oxidative insertion of iron model catalysts into $H_3C–X$ bonds (X = H, Cl, $CH_3$), computed at ZORA-OPBE/TZ2P.

**Table S4.** Electronic energies (in kcal $mol^{-1}$) relative to reactants for the oxidative insertion of $^1Fe(CO)_4$ into $H_3C–X$ bonds (X = H, Cl, $CH_3$) in the gas phase, computed at ZORA-OPBE/TZ2P, ZORA-BP86/TZ2P//ZORA-OPBE/TZ2P, ZORA-BLYP/TZ2P//ZORA-OPBE/TZ2P, ZORA-B3LYP/TZ2P//ZORA-OPBE/TZ2P, and ZORA-M06L/TZ2P//ZORA-OPBE/TZ2P.

**Table S5.** Geometry parameters (in Å, degrees) of anionic singlet $^1Fe(CO)_4^{-2}$, computed at ZORA-OPBE/TZ2P, ZORA-BLYP/TZ2P, ZORA-M06L/TZ2P, and from the literature.

**Table S6**. Geometry parameters (in Å, degrees) of singlet $^1Fe(CO)4$, computed at ZORA-OPBE/TZ2P, ZORA-BLYP/TZ2P, ZORA-M06L/TZ2P, and from the literature.

**Table S7**. Geometry parameters (in Å, degrees) of triplet $^3Fe(CO)4$, computed at ZORA-OPBE/TZ2P, ZORA-BLYP/TZ2P, ZORA-M06L/TZ2P, and from the literature.

**Fig. S1** Geometry and electronic energy (in kcal mol$^{-1}$) relative to reactants for the $S_N2$ reaction of anionic singlet $^1Fe(CO)_4^{-2}$ with $H_3C$–X bonds (X = H, Cl, $CH_3$), computed at ZORA-OPBE/TZ2P.

**Table S8**. Cartesian coordinates (in Å), ADF total bonding energies (in kcal mol$^{-1}$) and in parentheses, imaginary frequency of transition state structure (in cm$^{-1}$) of all stationary points in this study, computed at ZORA-OPBE/TZ2P.

**Table S1.** Electronic and, in parentheses, Gibbs free energies (in kcal $mol^{-1}$) relative to reactants for the oxidative insertion of iron model catalysts into $H_3C$–X bonds (X = H, Cl, $CH_3$), computed at ZORA-OPBE/TZ2P.

| | | **RC** | **TS** | **P** |
|---|---|---|---|---|
| $^{1}Fe(CO)_4$ | C–H | -1.3 (-0.8) | 10.4 (10.3) | 0.8 (2.3) |
| | C–Cl | -9.0 (-7.2) | 25.5 (27.6) | -15.1 (-11.3) |
| | C–C | 0.1 (0.2) | 48.0 (50.3) | 10.2 (12.4) |
| $^{3}Fe(CO)_4$ | C–H | 0 (0.1) | 64.4 (62.3) | 45.2 (49.0) |
| | C–Cl | 0 (0.2) | 43.3 (45.2) | 29.4 (30.8) |
| | C–C | -0.1 (-0.4) | 76.9 (75.8) | 51.1 (49.2) |
| $^{1}Fe(CO)_4^{-2}$ | C–H | -2.4 (-2.3) | 74.1 (69.8) | 66.3 (62.4) |

**Table S2.** Electronic energy with small frozen core and, in parentheses, electronic energy with no frozen core (in kcal $mol^{-1}$) for the oxidative insertion of iron model catalysts into $H_3C$–X bonds (X = H, Cl, $CH_3$), computed at ZORA-OPBE/TZ2P.

| | | RC | TS | P |
|---|---|---|---|---|
| $^1Fe(CO)_4$ | C–H | -1.3 (-1.6) | 10.4 (9.9) | 0.8 (1.4) |
| | C–Cl | -9.0 (-9.5) | 25.5 (24.9) | -15.1 (-15.8) |
| | C–C | 0.1 (0.2) | 48.0 (47.5) | 10.2 (9.8) |
| $^3Fe(CO)_4$ | C–H | 0 (0) | 64.4 (64.6) | 45.2 (46.4) |
| | C–Cl | 0 (0) | 43.3 (43.4) | 29.4 (29.6) |
| | C–C | -0.1 (0.2) | 76.9 (76.9) | 51.1 (50.9) |
| $^1Fe(CO)_4^{-2}$ | C–H | -2.4 (-2.1) | 74.1 (73.9) | 66.3 (66.4) |

**Table S3.** Electronic energy (in kcal mol$^{-1}$) in gas phase and, in parentheses, electronic energy in solution (THF) relative to reactants for the oxidative insertion of iron model catalysts into $H_3C$–X bonds (X = H, Cl, $CH_3$), computed at ZORA-OPBE/TZ2P.

| | | **RC** | **TS** | **P** |
|---|---|---|---|---|
| $^1Fe(CO)_4$ | C–H | -1.3 (-0.25) | 10.4 (11.2) | 0.8 (3.15) |
| | C–Cl | -9.0 (-7.8) | 25.5 (28.1) | -15.1 (-13.8) |
| | C–C | 0.1 (0.2) | 48.0 (49.1) | 10.2 (12.3) |
| $^3Fe(CO)_4$ | C–H | 0 (1.2) | 64.4 (64.3) | 45.2 (48.6) |
| | C–Cl | 0 (0.3) | 43.3 (43.4) | 29.4 (30.3) |
| | C–C | -0.1 (-0.7) | 76.9 (77.1) | 51.1 (51.3) |
| $^1Fe(CO)_4^{-2}$ | C–H | -2.4 (-1.3) | 74.1 (81.4) | 66.3 (69.4) |

**Table S4.** Electronic energies (in kcal mol$^{-1}$) relative to reactants for the oxidative insertion of $^{1}Fe(CO)_4$ into $H_3C$–X bonds (X = H, Cl, $CH_3$) in the gas phase, computed at ZORA-OPBE/TZ2P, ZORA-BP86/TZ2P//ZORA-OPBE/TZ2P, ZORA-BLYP/TZ2P//ZORA-OPBE/TZ2P, ZORA-B3LYP/TZ2P//ZORA-OPBE/TZ2P, and ZORA-M06L/TZ2P//ZORA-OPBE/TZ2P.

| | **Method** [a] | **RC** | **TS** | **P** |
|---|---|---|---|---|
| C–H | OPBE | -1.3 | 10.4 | 0.8 |
| | BP86 | -1.8 | 10.9 | 0.4 |
| | BLYP | -1.5 | 14.5 | 3.4 |
| | B3LYP | -2.2 | 14.4 | 2.3 |
| | M06-L | -5.3 | 9.6 | -1.1 |
| C–Cl | OPBE | -9.0 | 25.5 | -15.1 |
| | BP86 | -10.0 | 20.4 | -20.6 |
| | BLYP | -9.1 | 20.4 | -19.5 |
| | B3LYP | -10.2 | 23.4 | -22.2 |
| | M06-L | -8.2 | 22.8 | -24.3 |
| C–C | OPBE | 0.1 | 48.0 | 10.2 |
| | BP86 | -1.1 | 41.7 | 3.2 |
| | BLYP | -0.7 | 47.9 | 9.3 |
| | B3LYP | -1.0 | 50.9 | 9.8 |
| | M06-L | -1.2 | 45.9 | 5.9 |

[a] Indicates the DFT functional used to calculate the energy.

**Table S5.** Geometry parameters (in Å, degrees) of anionic singlet $^{1}Fe(CO)_4^{-2}$, computed at ZORA-OPBE/TZ2P, ZORA-BLYP/TZ2P, ZORA-M06L/TZ2P, and from the literature.

| Method | Fe – C | Fe - C | C – O | C - O | <(Fe – C – O) | <(Fe – C – O) | <(C – Fe – C) | <(C – Fe – C) |
|---|---|---|---|---|---|---|---|---|
| OPBE[a] | 1.727 | 1.727 | 1.200 | 1.200 | 179.8 | 179.8 | 109.7 | 109.7 |
| BLYP[a] | 1.771 | 1.771 | 1.206 | 1.206 | 179.9 | 179.9 | 109.6 | 109.6 |
| M06-L[a] | 1.747 | 1.747 | 1.194 | 1.194 | 179.9 | 179.9 | 109.6 | 109.6 |
| Exp[b] | 1.742 | 1.742 | 1.175 | 1.172 | - | - | 109.1 | 108.6 |
| Exp[c] | 1.738 | 1.745 | 1.175 | 1.162 | - | - | 109.5 | 109.6 |

[a] This work: computed using ZORA for reletivistic effects and the TZ2P basis set.
[b] R. G. Teller, R. G. Finke, J. P. Collman, H. B. Chin, R. Bau, *J. Am. Chem. Soc.* **1977**, *99*, 1104-1111.
Experimental results from X-ray crystal structures.
[c] H. B. Chin, R. Bau. J. Am. Chem. Soc. 1976, 98, 2434-2439.
Experimental results from X-ray crystal structures.

**Table S6.** Geometry parameters (in Å, degrees) of singlet $^1Fe(CO)_4$, computed at ZORA-OPBE/TZ2P, ZORA-BLYP/TZ2P, ZORA-M06L/TZ2P, and from the literature.

| **Method** | **Fe – C** | **Fe - C** | **C – O** | **C - O** | **<(Fe – C – O)** | **<(Fe – C – O)** | **<(C – Fe – C)** | **<(C – Fe – C)** |
|---|---|---|---|---|---|---|---|---|
| OPBE[a] | 1.777 | 1.732 | 1.151 | 1.158 | 178.2 | 170.5 | 181,7 | 126.6 |
| BLYP[a] | 1.806 | 1.806 | 1.157 | 1.157 | 170.3 | 170.3 | 141.5 | 141.8 |
| M06-L[a] | 1.808 | 1.773 | 1.144 | 1.149 | 172.1 | 170.5 | 154.4 | 129.6 |
| Exp[b] | 1.78 | 1.74 | - | - | - | - | 177.4 | 135.3 |
| BP86[c] | 1.803 | 1.770 | 1.153 | 1.153 | - | - | 159.8 | 130.2 |
| M06-L[d] | 1.823 | 1.777 | 1.144 | 1.151 | - | - | 168.3 | 127.8 |

[a] This work: computed using ZORA for reletivistic effects and the TZ2P basis set.
[b] H. Ihee, J. M. Cao, A. H. Zewail, *Angew. Chem. Int. Ed.* **2001**, *40*, 1532-1536. Experimental results from ultrafast electron diffraction.
[c] A. Krapp, K. K. Pandey, G. Frenking, *J. Am. Chem. Soc.* **2007**, *129*, 7596-7610. Computational results using ZORA for reletivistic effects and the TZ2P basis set.
[d] Z. Sun, H. F. Schaefer III, Y. Xie, Y. Liu, R. Zhong, *J. Comput. Chem.* **2014**, *35*, 998-1009. Computational results using cc-pVTZ basis set.

**Table S7.** Geometry parameters (in Å, degrees) of triplet $^3Fe(CO)_4$, computed at ZORA-OPBE/TZ2P, ZORA-BLYP/TZ2P, ZORA-M06L/TZ2P, and from the literature.

| Method | Fe – C | Fe - C | C – O | C - O | <(Fe – C – O) | <(Fe – C – O) | <(C – Fe – C) | <(C – Fe – C) |
|---|---|---|---|---|---|---|---|---|
| OPBE[a] | 1.810 | 1.761 | 1.151 | 1.153 | 175.8 | 178.8 | 154.5 | 96 |
| BLYP[a] | 1.863 | 1.827 | 1.153 | 1.155 | 178.7 | 179.5 | 146.3 | 98.5 |
| M06-L[a] | 1.855 | 1.826 | 1.142 | 1.145 | 179.6 | 179.9 | 144.7 | 98.3 |
| Exp[b] | - | - | - | - | - | - | 145 | 120 |
| BP86[c] | 1.803 | 1.770 | 1.153 | 1.153 | - | - | 159.8 | 130.2 |
| M06-L[d] | 1.869 | 1.840 | 1.143 | 1.146 | - | - | 146.4 | 97.4 |

[a] This work: computed using ZORA for reletivistic effects and the TZ2P basis set.
[b] H. Ihee, J. M. Cao, A. H. Zewail, *Angew. Chem. Int. Ed.* **2001**, *40*, 1532-1536. Experimental results from ultrafast electron diffraction.
[c] A. Krapp, K. K. Pandey, G. Frenking, *J. Am. Chem. Soc.* **2007**, *129*, 7596-7610. Computational results using ZORA for reletivistic effects and the TZ2P basis set.
[d] Z. Sun, H. F. Schaefer III, Y. Xie, Y. Liu, R. Zhong, *J. Comput. Chem.* **2014**, *35*, 998-1009. Computational results using cc-pVTZ basis set.

**Fig. S1** Geometry and electronic energy (in kcal mol$^{-1}$) relative to reactants for the $S_N2$ reaction of anionic singlet $^1Fe(CO)_4^{-2}$ with $H_3C–X$ bonds (where X = H, Cl, $CH_3$), computed at ZORA-OPBE/TZ2P.

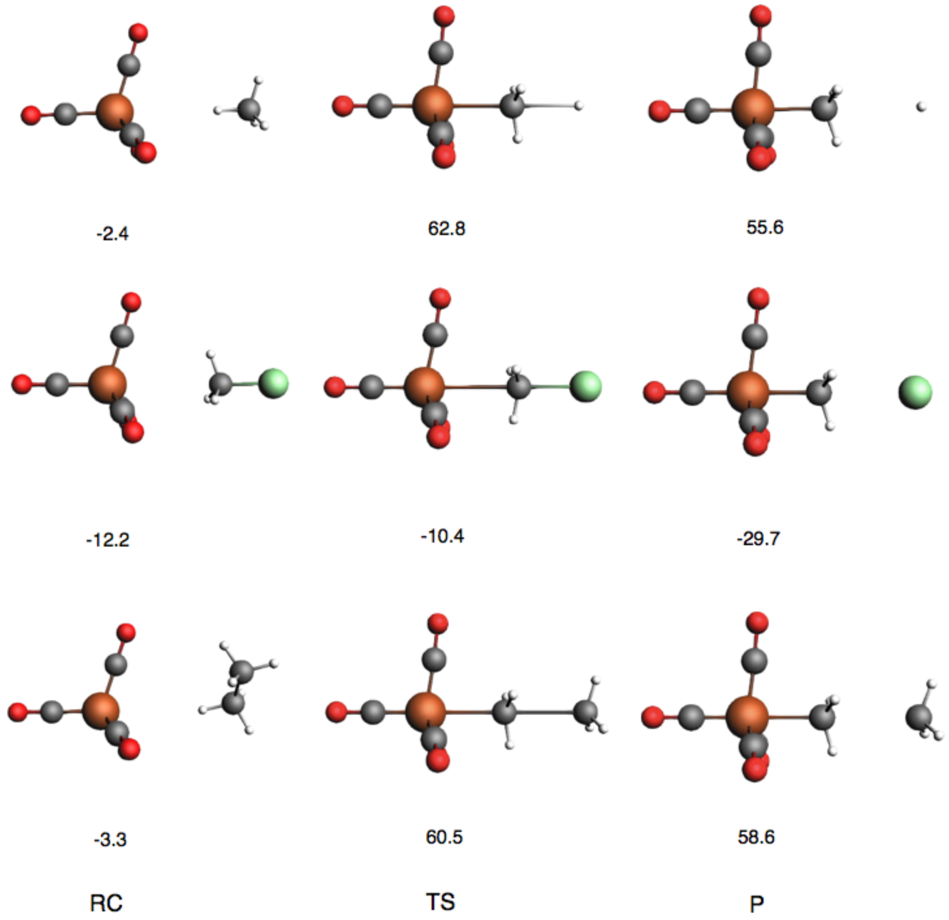

**Table S8.** Cartesian coordinates (in Å), ADF total bonding energies (in kcal mol$^{-1}$) and in parentheses, imaginary frequency of transition state structure (in cm$^{-1}$) of all stationary points in this study, computed at ZORA-OPBE/TZ2P.

RC: Pd+$CH_4$ -557.93

| | | | |
|---|---|---|---|
| C | 0.00000000 | 0.00000000 | -1.93543615 |
| Pd | 0.00000000 | 0.00000000 | 0.32205542 |
| H | -0.96181082 | 0.00000000 | -1.33610429 |
| H | 0.00000000 | -0.90063254 | -2.55201285 |
| H | 0.00000000 | 0.90063254 | -2.55201285 |
| H | 0.96181082 | 0.00000000 | -1.33610429 |

TS: Pd+$CH_4$ -548.86 (-638.52)

| | | | |
|---|---|---|---|
| C | -1.74196777 | -2.22087997 | 0.00000000 |
| Pd | -2.13750904 | -0.23784341 | 0.00000000 |
| H | -2.80956968 | -2.49954731 | 0.00000000 |
| H | -1.26528821 | -2.62993236 | 0.89567676 |
| H | -1.26528821 | -2.62993236 | -0.89567676 |
| H | -0.75509932 | -0.88569836 | 0.00000000 |

P: Pd+$CH_4$ -552.12

| | | | |
|---|---|---|---|
| C | -2.10134690 | -2.41901732 | 0.18620613 |
| Pd | -2.73145901 | -0.57025833 | 0.41934115 |
| H | -3.88639130 | -1.04648079 | -0.43099501 |
| H | -2.78392696 | -3.12497645 | 0.66994616 |
| H | -1.97386865 | -2.66955518 | -0.87144525 |
| H | -1.12556673 | -2.41201402 | 0.69858365 |

RC: Pd+$CH_3Cl$ -524.16

| | | | |
|---|---|---|---|
| C | -1.47287227 | -3.56288223 | 0.26327334 |
| Pd | -2.82627779 | -0.34245554 | 0.45624055 |
| H | -1.57461503 | -3.54757927 | -0.82290172 |
| H | -2.37930991 | -3.94569640 | 0.73456493 |
| H | -0.59964137 | -4.14645722 | 0.56552918 |
| Cl | -1.20791067 | -1.87762182 | 0.82999800 |

TS: Pd+$CH_3Cl$ -512.77 (-194.99)

| | | | |
|---|---|---|---|
| C | -2.17528160 | -2.70744274 | 0.04185559 |
| Pd | -2.81199527 | -0.48996192 | 0.39801798 |
| H | -3.03828060 | -2.14811751 | -0.44447729 |
| H | -2.57511176 | -3.48805558 | 0.68792144 |
| H | -1.56169511 | -3.12338103 | -0.75763162 |
| Cl | -0.93457054 | -1.80746229 | 1.11087980 |

P: Pd+$CH_3Cl$ -541.55

| | | | |
|---|---|---|---|
| C | -2.24128754 | -2.72324886 | 0.02797073 |
| Pd | -2.21746210 | -0.87254938 | 0.63669530 |
| Cl | -0.32812332 | -1.03352457 | 1.74551410 |
| H | -1.34236976 | -2.94908132 | -0.55095966 |
| H | -3.14585457 | -2.70484419 | -0.60465481 |
| H | -2.35050906 | -3.39492647 | 0.88372619 |

RC: Pd+$C_2H_6$ -936.92

| | | | |
|---|---|---|---|
| C | -0.88946600 | -2.04338800 | -0.17463600 |
| H | -2.49303200 | -3.37733700 | 0.43314500 |
| H | -1.23793300 | -2.00135900 | -1.25408600 |
| H | -0.08816400 | -2.78887200 | -0.15842300 |
| H | -0.40987200 | -1.07999400 | 0.18635900 |
| H | -1.72154400 | -2.51878800 | 1.77520600 |
| C | -2.05029400 | -2.41930700 | 0.73315900 |
| H | -2.84402800 | -1.66562800 | 0.70730000 |
| Pd | -0.64978700 | -0.18374000 | -1.50171800 |

TS: Pd+$C_2H_6$ -913.48 (-480.74)

| | | | |
|---|---|---|---|
| C | 0.70009173 | 0.65754447 | -1.57799445 |
| H | -0.20436740 | -1.36376803 | -0.91184594 |
| H | 1.22041679 | 1.28476466 | -2.36320542 |
| H | 1.47760465 | 0.10735848 | -1.04752662 |
| H | 0.20436740 | 1.36376803 | -0.91184594 |
| H | -1.47760465 | -0.10735848 | -1.04752662 |
| C | -0.70009173 | -0.65754447 | -1.57799445 |
| H | -1.22041679 | -1.28476466 | -2.36320542 |
| Pd | 0.00000000 | 0.00000000 | -3.39592210 |

P: Pd+$C_2H_6$ -934.44

| | | | |
|---|---|---|---|
| C | 1.44496927 | 0.00000000 | -1.98027211 |
| H | -1.40855357 | -0.90094754 | -1.35980537 |
| H | 2.35940354 | 0.00000000 | -2.59580903 |
| H | 1.40855357 | -0.90094754 | -1.35980537 |
| H | 1.40855357 | 0.90094754 | -1.35980537 |
| H | -1.40855357 | 0.90094754 | -1.35980537 |
| C | -1.44496927 | 0.00000000 | -1.98027211 |
| H | -2.35940354 | 0.00000000 | -2.59580903 |
| Pd | 0.00000000 | 0.00000000 | -3.31857216 |

RC: $Pd(CO)_2$+ $CH_4$ -1329.61

| | | | |
|---|---|---|---|
| Pd | -0.06414951 | -2.44190500 | 1.79987105 |
| H | 2.50988985 | -7.35497188 | -0.25450190 |
| H | 0.94140707 | -6.51790215 | -0.09782246 |
| C | -1.85275106 | -1.81378783 | 1.62990427 |
| C | 1.94529528 | -6.45940250 | -0.52651035 |
| H | 1.87202436 | -6.39201982 | -1.61454559 |
| C | 1.58542910 | -2.11550416 | 2.69199056 |
| H | 2.45913377 | -5.57308254 | -0.14137475 |
| O | -2.87858188 | -1.29892266 | 1.65930362 |
| O | 2.48393881 | -1.76824590 | 3.31675104 |

TS: $Pd(CO)_2$+ $CH_4$ -1296.17 (-542.12)

| | | | |
|---|---|---|---|
| Pd | 0.29344087 | -3.40955739 | 1.17923774 |
| H | -1.61529078 | -3.27886712 | -0.58090923 |
| H | 0.35365903 | -4.68173609 | 0.27044156 |
| C | -0.49795146 | -1.83188013 | 2.07464044 |
| C | -1.35176932 | -4.13143809 | 0.05415290 |
| H | -1.30483947 | -5.02385777 | -0.57157505 |
| C | 2.12156276 | -3.51852731 | 1.78019728 |
| H | -2.12722996 | -4.28315103 | 0.81257110 |
| O | -0.93770215 | -0.92497230 | 2.61691378 |
| O | 3.21453376 | -3.67518078 | 2.08730242 |

P: $Pd(CO)_2$+ $CH_4$ -1297.91

| | | | |
|---|---|---|---|
| Pd | 0.02532863 | -2.30155893 | 1.95129640 |
| C | 1.56954094 | -1.72674987 | 2.94150840 |
| C | -1.41905751 | -1.06129709 | 2.41841636 |
| O | 2.55914852 | -1.50099073 | 3.47022442 |
| O | -2.29199261 | -0.36623478 | 2.66362953 |
| H | 0.96480554 | -3.43413170 | 1.39982931 |
| C | -1.23322298 | -3.37235891 | 0.68304309 |
| H | -0.76158454 | -4.22944304 | 0.20427162 |
| H | -1.59080701 | -2.68049396 | -0.08811253 |
| H | -2.08788925 | -3.71655134 | 1.27651445 |

RC: $Pd(CO)_2$+ $CH_3Cl$ -1292.30

| | | | |
|---|---|---|---|
| Pd | 0.20762769 | -2.17082175 | 3.45685133 |
| H | -1.96339015 | 1.00868608 | 1.78909139 |
| H | -2.87381105 | 1.82609219 | 0.48098263 |
| C | -1.25531376 | -3.27950337 | 2.94676826 |
| C | -2.51130688 | 1.91041479 | 1.50779981 |
| H | -1.87020167 | 2.78876919 | 1.61002360 |
| C | 1.42952685 | -0.86482051 | 2.80934747 |
| Cl | -3.90095569 | 2.09114745 | 2.58784157 |
| O | -2.11244291 | -3.86990338 | 2.46292836 |
| O | 2.07066594 | -0.09551138 | 2.24721489 |

TS: $Pd(CO)_2$+ $CH_3Cl$ -1257.35 (-287.64)

| | | | |
|---|---|---|---|
| Pd | 0.09340518 | -3.40057459 | 1.33616957 |
| O | -2.47799499 | -3.82055043 | -0.34278022 |
| O | 0.67086432 | -1.18500901 | 3.42582258 |
| C | -1.52373170 | -3.71131258 | 0.28594105 |
| C | 0.47886335 | -2.04302150 | 2.68773539 |
| Cl | 1.79684075 | -4.98498591 | 1.01151774 |
| C | 1.65123748 | -3.06102446 | -0.20645330 |
| H | 2.65656415 | -2.82493719 | 0.12690288 |
| H | 1.00886289 | -2.16895922 | -0.27920806 |
| H | 1.61701657 | -3.67501512 | -1.10052938 |

P: $Pd(CO)_2$+ $CH_3Cl$ -1287.03

| | | | |
|---|---|---|---|
| Pd | -0.06557000 | -2.36175000 | 1.97496900 |
| C | 1.47139900 | -1.93311300 | 3.28758400 |
| C | -1.01001800 | -0.69873700 | 1.90206300 |
| O | 2.35767000 | -1.81288700 | 3.99473200 |
| O | -1.59562200 | 0.28656400 | 1.83970400 |
| Cl | 0.90205200 | -4.50890400 | 1.88015000 |
| C | -1.51668900 | -3.09251400 | 0.60904700 |
| H | -0.92324900 | -3.41743600 | -0.24956100 |
| H | -2.27125900 | -2.35535900 | 0.32396600 |
| H | -1.95968700 | -3.94371400 | 1.13269400 |

RC: $Pd(CO)_2$+ +$C_2H_6$ -1709.10

| | | | |
|---|---|---|---|
| Pd | -0.57878086 | -2.82371036 | 1.31950782 |
| O | -3.05617499 | -3.63780186 | -0.24253754 |
| O | 0.12855578 | -0.50430888 | 3.15302006 |
| C | -2.06469166 | -3.43560307 | 0.30034990 |
| C | -0.02035174 | -1.42367619 | 2.48135918 |
| H | 4.81671409 | -7.24622468 | 1.36478756 |
| C | 3.37849241 | -5.78123859 | 0.64709165 |
| H | 3.56083128 | -5.06099426 | 1.45334440 |
| H | 2.49734990 | -6.36592884 | 0.92701118 |
| H | 3.13041336 | -5.20889843 | -0.25457171 |
| C | 4.58434996 | -6.68428184 | 0.45279307 |
| H | 4.41443297 | -7.41413060 | -0.34726515 |
| H | 5.47819385 | -6.10842777 | 0.18615746 |

TS: $Pd(CO)_2$+ +$C_2H_6$ -1655.09 (-452.83)

| | | | |
|---|---|---|---|
| Pd | 0.07341342 | -3.28415047 | 1.22376583 |
| O | -2.65800856 | -3.41021992 | -0.18534767 |
| O | -0.14008205 | -0.93411461 | 3.20988952 |
| C | -1.68988159 | -3.54957793 | 0.41954986 |
| C | 0.04189081 | -1.72793607 | 2.39837514 |
| H | 1.97792841 | -5.52541075 | 1.45458490 |
| C | 2.13831009 | -3.46725921 | 0.63355276 |
| H | 2.81723182 | -3.38898705 | 1.48317521 |
| H | 1.96516206 | -2.47130769 | 0.19046166 |
| H | 2.56395305 | -4.09921054 | -0.14447344 |
| C | 1.03265356 | -5.19942006 | 1.02311526 |
| H | 0.22903779 | -5.51731315 | 1.71148837 |
| H | 0.88185066 | -5.64382216 | 0.03894981 |

P: $Pd(CO)_2$+ +$C_2H_6$ -1676.17

| | | | |
|---|---|---|---|
| Pd | 0.11506207 | -3.22483377 | 1.18544393 |
| O | -2.70867942 | -3.66055704 | 0.06241640 |
| O | -0.17841338 | -0.84934519 | 3.10760237 |
| C | -1.66875111 | -3.46473053 | 0.49665295 |
| C | -0.11144807 | -1.73352519 | 2.38497870 |
| C | 2.12915259 | -3.22598774 | 1.73243590 |
| C | 0.68991604 | -4.83729516 | -0.00703551 |
| H | 2.67310990 | -2.71490586 | 0.92996631 |
| H | 2.48113566 | -4.25733028 | 1.80647597 |
| H | 2.32321673 | -2.70980285 | 2.67681289 |
| H | 1.69359164 | -4.65841346 | -0.39870130 |
| H | 0.00535001 | -5.02513655 | -0.83899231 |
| H | 0.70756156 | -5.71842681 | 0.64459109 |

## RC: $Pd(PH_3)_2$+ $CH_4$

-1339.52

| | | | |
|---|---|---|---|
| Pd | 0.04601830 | -2.17968359 | 1.98078367 |
| H | 3.04935852 | -1.34955766 | 2.90448089 |
| H | -2.25553764 | -2.82680773 | -0.13477425 |
| P | -2.03178194 | -2.35516281 | 1.18972535 |
| H | -2.98230774 | -3.22812389 | 1.79285356 |
| H | -2.93808350 | -1.25937367 | 1.07843852 |
| P | 2.20359375 | -2.42092099 | 2.49040646 |
| H | 2.62874432 | -3.30612896 | 3.52252855 |
| H | 3.11567765 | -2.92003506 | 1.51784092 |
| C | -0.17876482 | -7.63492444 | 0.86100690 |
| H | -0.96466219 | -8.03168755 | 1.50879408 |
| H | -0.46352749 | -7.77611312 | -0.18482110 |
| H | -0.04343704 | -6.56937885 | 1.06222047 |
| H | 0.75633303 | -8.16557151 | 1.05757931 |

## TS: $Pd(PH_3)_2$+ $CH_4$

-1308.79 (-587.74)

| | | | |
|---|---|---|---|
| Pd | 0.14041712 | -2.99250931 | 1.74548279 |
| H | 2.34356608 | -1.95764203 | 3.82935674 |
| H | -2.95603752 | -2.02833049 | 0.97709791 |
| P | -1.69462983 | -1.59697923 | 1.48815767 |
| H | -2.25560925 | -0.90947861 | 2.60391396 |
| H | -1.63163215 | -0.43534195 | 0.66414927 |
| P | 1.74789922 | -1.63669753 | 2.57652182 |
| H | 2.98646166 | -1.51906151 | 1.88391945 |
| H | 1.65009088 | -0.23945252 | 2.85905753 |
| C | -0.55760979 | -4.88285714 | 1.08981960 |
| H | 0.03561137 | -5.80026809 | 1.08459367 |
| H | -0.87785051 | -4.68275084 | 0.06071308 |
| H | -1.43364795 | -5.05225318 | 1.72660068 |
| H | 0.99341583 | -4.30166428 | 1.71775803 |

## P: $Pd(PH_3)_2$+ $CH_4$

-1311.18

| | | | |
|---|---|---|---|
| Pd | 0.15847936 | -2.33035879 | 1.57912196 |
| H | 1.52238550 | 0.24518024 | 3.15660713 |
| H | -2.21628662 | -0.73479864 | 0.10615410 |
| P | -1.68592078 | -0.99178859 | 1.40052549 |
| H | -2.90946495 | -1.44552465 | 1.96647672 |
| H | -1.80601672 | 0.34991450 | 1.86903830 |
| P | 1.68478871 | -1.09180346 | 2.68082581 |
| H | 2.22278754 | -1.59940069 | 3.89482808 |
| H | 2.94002138 | -0.85785127 | 2.05611669 |
| H | 1.28582037 | -3.43474265 | 1.57241926 |
| C | -0.79769670 | -3.87753728 | 0.56085952 |
| H | -0.25263271 | -4.08239478 | -0.36675903 |
| H | -1.83769914 | -3.63832494 | 0.30955190 |
| H | -0.78732127 | -4.78478333 | 1.17328578 |

## RC: $Pd(PH_3)_2$+ $CH_3Cl$

-1302.27

| | | | |
|---|---|---|---|
| C | 3.39463592 | 1.62145001 | 0.25637816 |
| Cl | 3.83065213 | 3.21367138 | -0.38198811 |
| Pd | -1.22822534 | -0.61556209 | 0.05606666 |
| H | 2.30723846 | 1.52388909 | 0.24990551 |
| H | 3.84554256 | 0.85449122 | -0.37726890 |
| H | 3.77544516 | 1.53732085 | 1.27661011 |
| P | -0.80206013 | -0.47017285 | -2.12927737 |
| P | -1.77580393 | -0.29893513 | 2.19519817 |
| H | -1.57511035 | 0.97040364 | 2.80777217 |
| H | -1.81923691 | -0.70632779 | -3.09838547 |
| H | -3.12112424 | -0.44009661 | 2.64177744 |
| H | -1.21727002 | -1.04130417 | 3.27720280 |
| H | -0.37498258 | 0.76382895 | -2.69636949 |
| H | 0.18094802 | -1.25499649 | -2.80114727 |

## TS: $Pd(PH_3)_2$+ $CH_3Cl$

-1271.14 (-364.82)

| | | | |
|---|---|---|---|
| Pd | 0.08805989 | -2.99629170 | 1.85901762 |
| H | 2.73257805 | -2.30878568 | 3.81988686 |
| H | -1.41596857 | -0.32043439 | 0.66326354 |
| P | -1.62285286 | -1.68040974 | 1.03918255 |
| H | -2.40654213 | -1.97818175 | -0.11760734 |
| H | -2.75312452 | -1.37871435 | 1.85548273 |
| P | 1.66955674 | -1.80766544 | 3.00189027 |
| H | 2.52856524 | -0.97832415 | 2.22364005 |
| H | 1.34404767 | -0.76813692 | 3.92413852 |
| C | 0.29666168 | -5.06236222 | 2.69171151 |
| H | 1.32784901 | -5.31868036 | 2.92069548 |
| H | -0.34529285 | -5.93212602 | 2.58137260 |
| H | -0.15364876 | -4.37100803 | 3.43909941 |
| Cl | 0.38553501 | -4.94900827 | 0.55955361 |

## P: $Pd(PH_3)_2$+ $CH_3Cl$

-1309.76

| | | | |
|---|---|---|---|
| Pd | -0.04902266 | -2.39283936 | 1.69211277 |
| H | 1.70817573 | 0.14576356 | 2.99410678 |
| H | -1.62357836 | -0.69640822 | -0.39334794 |
| P | -1.39146549 | -0.83001254 | 1.00069659 |
| H | -2.75052381 | -0.86589242 | 1.40877646 |
| H | -1.15911321 | 0.54718105 | 1.27489373 |
| P | 1.62165107 | -1.26881885 | 2.81917210 |
| H | 1.82767774 | -1.62382693 | 4.17488513 |
| H | 2.94646029 | -1.46806442 | 2.35930493 |
| Cl | 1.23245250 | -4.23769250 | 2.31985006 |
| C | -1.40586688 | -3.64033214 | 0.74846575 |
| H | -0.79867758 | -4.20631115 | 0.03629264 |
| H | -2.25875991 | -3.18927123 | 0.22853059 |
| H | -1.76663589 | -4.29792989 | 1.54472788 |

## RC: $Pd(PH_3)_2$+ $C_2H_6$

-1718.93

| | | | |
|---|---|---|---|
| Pd | 0.00000000 | 0.00000000 | -1.15018261 |
| C | -0.23797370 | 0.72135286 | 4.29930754 |
| C | 0.23797370 | -0.72135286 | 4.29930754 |
| H | 0.11749259 | 1.26007901 | 3.41346394 |
| H | -1.33268182 | 0.78228736 | 4.30239499 |
| H | 0.12215960 | 1.26396554 | 5.18128127 |
| H | -0.11749259 | -1.26007901 | 3.41346394 |
| H | 1.33268182 | -0.78228736 | 4.30239499 |
| H | -0.12215960 | -1.26396554 | 5.18128127 |
| P | -1.82347349 | 1.25832773 | -0.89583284 |
| P | 1.82347349 | -1.25832773 | -0.89583284 |
| H | 1.76777675 | -2.47707469 | -0.16119136 |
| H | -2.57631227 | 1.80916731 | -1.97502850 |
| H | 2.57631227 | -1.80916731 | -1.97502850 |
| H | 2.96309159 | -0.75206817 | -0.20801902 |
| H | -2.96309159 | 0.75206817 | -0.20801902 |
| H | -1.76777675 | 2.47707469 | -0.16119136 |

## TS: $Pd(PH_3)_2$+ $C_2H_6$

-1666.99 (-446.72)

| | | | |
|---|---|---|---|
| Pd | 0.00513108 | -2.97517596 | 1.76589892 |
| H | 2.16587900 | -0.45395078 | 1.52675469 |
| H | -1.93854727 | -0.62402673 | 0.74366435 |
| P | -1.72998976 | -1.46468249 | 1.87707474 |
| H | -3.12179517 | -1.76918203 | 2.02272969 |
| H | -1.79627345 | -0.37752686 | 2.80176662 |
| P | 1.93308592 | -1.75371163 | 2.07253628 |
| H | 2.23087358 | -1.33996676 | 3.40487355 |
| H | 3.27322119 | -2.18454535 | 1.80876910 |
| C | -0.82948679 | -4.88514642 | 2.22901358 |
| C | 0.57829346 | -4.76630417 | 0.75493440 |
| H | 0.87313622 | -4.03435265 | -0.02278811 |
| H | -0.03860530 | -5.51785644 | 0.26043317 |
| H | 1.46723746 | -5.23219913 | 1.18418867 |
| H | -0.33259305 | -5.82528190 | 2.47233963 |
| H | -1.76544676 | -5.07974156 | 1.70226832 |
| H | -1.03573801 | -4.37624316 | 3.19107997 |

## P: $Pd(PH_3)_2$+ $C_2H_6$

-1688.89

| | | | |
|---|---|---|---|
| Pd | 0.02031695 | -2.52480198 | 1.96738709 |
| H | 1.56518650 | -0.20191873 | 3.74153899 |
| H | -2.39458930 | -0.66166249 | 1.00256823 |
| P | -1.67423405 | -1.04319883 | 2.16770896 |
| H | -2.81766978 | -1.42062926 | 2.92548554 |
| H | -1.57761919 | 0.27231846 | 2.71146912 |
| P | 1.68516945 | -1.44035091 | 3.04323853 |
| H | 2.38701138 | -2.11793001 | 4.07789442 |
| H | 2.84276619 | -1.06979555 | 2.30397087 |
| C | 1.37305133 | -4.08373651 | 1.65599777 |
| C | -1.30271708 | -3.74139899 | 0.90641420 |
| H | -1.07688281 | -3.60996414 | -0.15878806 |
| H | -2.36259560 | -3.50733985 | 1.06745082 |
| H | -1.13597553 | -4.78750521 | 1.17829400 |
| H | 1.24625538 | -4.47668890 | 0.64311351 |
| H | 1.12787572 | -4.87257536 | 2.37722479 |
| H | 2.42640396 | -3.80700786 | 1.78983268 |

## RC: $Pd(PH_3)_2CH_3Cl$ + $MgCH_3Cl$

-1890.91

| | | | |
|---|---|---|---|
| H | 0.04681000 | -2.73421600 | 3.70857300 |
| Pd | -0.57754500 | -4.31546600 | 1.07736500 |
| H | -2.32976000 | -1.99809100 | -0.55322000 |
| H | -1.98297100 | -2.37104700 | 3.17482000 |
| C | 0.24684000 | -5.36985200 | 2.64977000 |
| P | -1.61314800 | -3.21363400 | -0.70358000 |
| H | -0.84098800 | -2.81768300 | -1.82315200 |
| H | -2.61250000 | -3.92761900 | -1.40631600 |
| P | -0.75874000 | -2.71538200 | 2.54651000 |
| H | -0.42894500 | -1.41090300 | 2.09997900 |
| H | 1.28110000 | -5.03763900 | 2.78330700 |
| H | 0.21757800 | -6.40945500 | 2.31735200 |
| H | -0.31008100 | -5.25742700 | 3.58465600 |
| Cl | -0.01831300 | -6.06716700 | -0.42618800 |
| Mg | 2.15530000 | -5.32556600 | -1.49363300 |
| Cl | 2.28070800 | -6.38938900 | -3.47054000 |
| C | 3.23133200 | -3.82222700 | -0.46968600 |
| H | 3.41953400 | -4.03194200 | 0.59380100 |
| H | 2.77601700 | -2.82151600 | -0.51524300 |
| H | 4.22101800 | -3.73180400 | -0.94313200 |

## TS: $Pd(PH_3)_2CH_3Cl$ + $MgCH_3Cl$

-1886.06 (-94.38)

| | | | |
|---|---|---|---|
| H | 0.22494600 | -2.72581500 | 3.58882500 |
| Pd | -0.29940700 | -4.21042600 | 0.90140200 |
| H | -1.44811900 | -1.49780700 | -0.54645500 |
| H | -1.63823500 | -3.76112400 | 3.67626000 |
| C | 0.64143600 | -5.40866700 | 2.31212800 |
| P | -1.43854200 | -2.91355200 | -0.64104700 |
| H | -1.14590600 | -2.99491200 | -2.02329200 |
| H | -2.84120200 | -3.08501800 | -0.74798300 |
| P | -0.80877700 | -3.13715500 | 2.71248300 |
| H | -1.50101200 | -1.89872400 | 2.65223000 |
| H | 1.69966500 | -5.13483300 | 2.28155600 |
| H | 0.47008000 | -6.41624800 | 1.92567500 |
| H | 0.28661300 | -5.36953500 | 3.34784800 |
| Cl | -0.62730100 | -6.16752400 | -0.66582700 |
| Mg | 1.52001900 | -5.41478600 | -1.34603700 |
| Cl | 2.68181300 | -6.50607700 | -2.91541600 |
| C | 2.12040400 | -3.57954400 | -0.39790800 |
| H | 3.11723200 | -3.89191600 | -0.04501700 |
| H | 1.75015600 | -2.89321600 | 0.37379900 |
| H | 2.28830300 | -2.95114000 | -1.28798400 |

## P: $Pd(PH_3)_2CH_3Cl$ + $MgCH_3Cl$

-1907.77

| | | | |
|---|---|---|---|
| Pd | -0.06374500 | 0.94031200 | 0.16194700 |
| C | -1.41350600 | 1.16880700 | 1.72486200 |
| H | -0.78581900 | -1.93248500 | -1.27298700 |
| Cl | 3.26808000 | -0.10425800 | 1.23700100 |
| H | 0.45638800 | 3.24660100 | 1.64372800 |
| H | 2.58971900 | 1.59018700 | -1.63581400 |
| Mg | 1.70061100 | 1.20550500 | 2.21488100 |
| Cl | 1.46287500 | 1.97959000 | 4.29617300 |
| C | 0.66992100 | 2.84782800 | 0.64321800 |
| H | 1.71641900 | 3.05830600 | 0.38130100 |
| P | 1.43437000 | 0.77828800 | -1.59361400 |
| H | 0.05531800 | 3.44073600 | -0.04734800 |
| H | 2.04637800 | -0.45741000 | -1.90264200 |
| P | -1.00623900 | -1.10596000 | -0.14036500 |
| H | -2.41756000 | -1.22571300 | -0.10753400 |
| H | -0.71470800 | -2.08125700 | 0.84291900 |
| H | 0.95888900 | 1.07013200 | -2.90212600 |
| H | -1.74177600 | 0.23004400 | 2.18491100 |
| H | -2.27416900 | 1.63527600 | 1.22667700 |
| H | -1.07062900 | 1.83338200 | 2.52100700 |

## RC: $^{1}Fe(CO)_4$ +$CH_4$ -2211.23

| | | | |
|---|---|---|---|
| Fe | 0.35780891 | 0.03825438 | 0.48752113 |
| O | -1.43802173 | -1.12505914 | -1.45217119 |
| C | -0.66267593 | -0.72030439 | -0.69282048 |
| O | -0.42629797 | 2.40938870 | 1.94586288 |
| O | 1.31561351 | 1.80643037 | -1.63395533 |
| O | -1.02975892 | -1.52409692 | 2.53166218 |
| C | -0.45657654 | -0.92268086 | 1.73313339 |
| C | 0.96585099 | 1.09867994 | -0.79455246 |
| C | -0.03387049 | 1.46193516 | 1.40712795 |
| C | 2.63288174 | -0.90589212 | 1.00999077 |
| H | 3.49023136 | -0.75012839 | 0.35348151 |
| H | 2.76932847 | -1.78794449 | 1.63748323 |
| H | 2.49816316 | -0.02749979 | 1.64450324 |
| H | 1.77347037 | -1.16072056 | 0.32248459 |

## TS: $^{1}Fe(CO)_4$ +$CH_4$ -2199.50 (-751.90)

| | | | |
|---|---|---|---|
| Fe | 0.34088938 | 0.01304017 | 0.46047141 |
| O | -1.75076143 | -0.80080398 | -1.37625394 |
| C | -0.92335517 | -0.47287970 | -0.64254083 |
| O | -0.49825553 | 2.41831727 | 1.90230645 |
| O | 1.69917876 | 1.48348141 | -1.65721901 |
| O | -0.63146929 | -1.83766027 | 2.49088409 |
| C | -0.26352657 | -1.09418265 | 1.69324896 |
| C | 1.14917883 | 0.91693750 | -0.82001466 |
| C | -0.16094089 | 1.47030656 | 1.33983353 |
| C | 2.30114584 | -0.40453727 | 1.22610154 |
| H | 3.11113121 | -0.34009600 | 0.49583177 |
| H | 2.39620583 | -1.34546235 | 1.77286522 |
| H | 2.39720271 | 0.42297797 | 1.93088228 |
| H | 1.34784361 | -1.05951162 | 0.16991896 |

## P: $^{1}Fe(CO)_4$ +$CH_4$ -2208.92

| | | | |
|---|---|---|---|
| Fe | 0.29982211 | -0.00309893 | 0.42404388 |
| O | -2.07990493 | -0.37020876 | -1.20397145 |
| C | -1.13897130 | -0.22101296 | -0.55807796 |
| O | -0.56545804 | 2.40898995 | 1.83351285 |
| O | 2.02676697 | 1.02740617 | -1.66830284 |
| O | -0.22973443 | -2.13374035 | 2.32203604 |
| C | -0.05907275 | -1.26273353 | 1.59062569 |
| C | 1.31518802 | 0.66254168 | -0.84171635 |
| C | -0.23953997 | 1.45977257 | 1.27227726 |
| C | 2.07286263 | 0.15490377 | 1.54879572 |
| H | 2.55967332 | 1.11154984 | 1.34507853 |
| H | 2.73999583 | -0.66394612 | 1.27566563 |
| H | 1.84296859 | 0.10398148 | 2.61564658 |
| H | 0.91467813 | -1.22524559 | -0.19518736 |

## RC: $^{1}Fe(CO)_4$ +$CH_3Cl$ -2181.41

| | | | |
|---|---|---|---|
| Fe | -0.01936064 | -0.15862999 | -0.26387674 |
| O | -0.05035324 | -0.34955885 | -3.14878138 |
| C | -0.02973485 | -0.20042048 | -2.00013474 |
| O | -0.28789361 | -2.56497274 | 1.31632131 |
| O | -2.93782201 | -0.03451368 | -0.35282162 |
| O | 2.85057573 | -0.69917106 | -0.37188667 |
| C | 1.72388824 | -0.45826832 | -0.31687186 |
| C | -1.78551936 | -0.05560673 | -0.30540754 |
| C | -0.17602063 | -1.56121316 | 0.74657031 |
| C | 0.23109620 | 1.97471388 | 2.43450193 |
| H | 0.35118661 | 3.01126695 | 2.75725060 |
| H | 1.06653635 | 1.35913434 | 2.76977676 |
| H | -0.72146230 | 1.56535314 | 2.77266635 |
| Cl | 0.22986139 | 1.98869686 | 0.64936339 |

## TS: $^{1}Fe(CO)_4$ +$CH_3Cl$ -2146.98 (-495.43)

| | | | |
|---|---|---|---|
| Fe | -0.01429889 | -0.11269874 | -0.13857699 |
| O | -0.06852218 | -0.49641017 | -3.02548199 |
| C | -0.03596406 | -0.25567690 | -1.89807899 |
| O | -0.33404494 | -2.82228609 | 0.79166232 |
| O | -2.91481956 | 0.23119949 | 0.08394145 |
| O | 2.88603516 | -0.46463908 | 0.07135333 |
| C | 1.74573416 | -0.31735114 | 0.00222885 |
| C | -1.77238968 | 0.10424892 | 0.00992306 |
| C | -0.20459094 | -1.72268538 | 0.45468050 |
| C | 0.14161184 | 1.15746084 | 2.12213979 |
| H | -0.69828766 | 1.77643887 | 2.42327756 |
| H | 1.11104070 | 1.54760304 | 2.41749445 |
| H | 0.01015151 | 0.11363674 | 2.37992803 |
| Cl | 0.26296998 | 2.19488517 | 0.21572376 |

## P: $^{1}Fe(CO)_4$ +$CH_3Cl$ -2187.36

| | | | |
|---|---|---|---|
| Fe | 0.33093818 | -0.02881487 | 0.42087429 |
| O | -2.07971223 | -0.36596849 | -1.20104467 |
| C | -1.13011226 | -0.24308374 | -0.57146966 |
| O | -0.55415038 | 2.34501538 | 1.79235124 |
| O | 1.95097857 | 1.38156260 | -1.56368296 |
| O | -0.38352240 | -1.88404422 | 2.56541825 |
| C | -0.13022520 | -1.15219512 | 1.72318340 |
| C | 1.29477969 | 0.84115422 | -0.79786029 |
| C | -0.20994704 | 1.39624218 | 1.23977484 |
| C | 2.06989184 | 0.18331183 | 1.57217398 |
| H | 2.56501440 | 1.13675228 | 1.37234080 |
| H | 2.69916884 | -0.65242942 | 1.26316322 |
| H | 1.84822299 | 0.12773522 | 2.64077392 |
| Cl | 1.18469389 | -1.91172218 | -0.58711381 |

## RC: $^{1}Fe(CO)_4$ + $C_2H_6$ -2589.31

| | | | |
|---|---|---|---|
| Fe | -0.04757578 | 0.12197323 | 0.39976386 |
| O | -2.05325228 | -0.66088348 | -1.51528489 |
| C | -1.18535077 | -0.43464220 | -0.78187352 |
| O | -0.50757653 | 2.43697079 | 2.05278637 |
| O | 1.13340471 | 1.87593406 | -1.62519506 |
| O | -1.41089828 | -1.51241064 | 2.40975412 |
| C | -0.85371760 | -0.88414754 | 1.62202266 |
| C | 0.68936189 | 1.17380734 | -0.82802158 |
| C | -0.22338940 | 1.49467208 | 1.44152169 |
| C | 4.12323181 | -2.01965385 | 0.77444093 |
| H | 4.37797617 | -2.91775412 | 0.19959108 |
| H | 4.77527639 | -1.99747048 | 1.65551947 |
| H | 4.37806942 | -1.15095074 | 0.15590036 |
| C | 2.65555387 | -2.01310953 | 1.17107700 |
| H | 2.40526372 | -1.11480739 | 1.74956176 |
| H | 2.40111484 | -2.88039174 | 1.79176817 |
| H | 2.00501656 | -2.04441900 | 0.28792820 |

## TS: $^{1}Fe(CO)_4$ + $C_2H_6$ -2541.36 (-621.71)

| | | | |
|---|---|---|---|
| Fe | -0.00983488 | 0.17516124 | -0.08517160 |
| O | 0.03977797 | -2.41684263 | -1.37533315 |
| C | 0.01829803 | -1.35001954 | -0.92516537 |
| O | 0.04963446 | -0.44037329 | 2.74791143 |
| O | -2.93023373 | 0.06562753 | -0.15108061 |
| O | 2.90979022 | 0.22306481 | -0.22321677 |
| C | 1.75732761 | 0.21116134 | -0.17200543 |
| C | -1.77794365 | 0.11633342 | -0.12703553 |
| C | 0.02439530 | -0.13155813 | 1.63377413 |
| C | -0.06802213 | 2.44617394 | 0.15844692 |
| H | -0.93305138 | 2.39110789 | 0.81850167 |
| H | -0.14124653 | 3.38285705 | -0.39280861 |
| H | 0.85534800 | 2.47551268 | 0.73584725 |
| C | -0.06928757 | 1.74877952 | -1.70370198 |
| H | 0.80092663 | 2.35803051 | -1.94793869 |
| H | -0.99272043 | 2.27761731 | -1.93855186 |
| H | -0.03075773 | 0.85424487 | -2.32574463 |

## P: $^{1}Fe(CO)_4$ + $C_2H_6$ -2578.97

| | | | |
|---|---|---|---|
| Fe | 0.32945709 | -0.05469896 | 0.40141263 |
| O | -2.07842475 | -0.38647333 | -1.19443617 |
| C | -1.12452707 | -0.25479792 | -0.56346469 |
| O | -0.57921349 | 2.34363627 | 1.77100097 |
| O | 2.02402957 | 1.20595415 | -1.59763694 |
| O | -0.25456433 | -1.99979697 | 2.48286431 |
| C | -0.05513131 | -1.21808069 | 1.66329281 |
| C | 1.32854932 | 0.72875454 | -0.81588334 |
| C | -0.22171034 | 1.39346745 | 1.22810678 |
| C | 2.09186231 | 0.13861601 | 1.53626919 |
| H | 2.57838291 | 1.09609337 | 1.33285040 |
| H | 2.76410824 | -0.67845192 | 1.26900014 |
| H | 1.86838686 | 0.09567192 | 2.60540678 |
| C | 1.04552014 | -1.78562933 | -0.55873600 |
| H | 2.04297912 | -2.00467554 | -0.17386477 |
| H | 1.09431879 | -1.63433301 | -1.64021001 |
| H | 0.38366888 | -2.63432263 | -0.36763315 |

## RC: $^{3}Fe(CO)_4$ + $CH_4$ -2209.92

| | | | |
|---|---|---|---|
| Fe | -0.36045477 | 1.44503040 | 0.16561190 |
| O | -3.17435394 | 0.64507153 | -0.28145876 |
| C | -2.06402726 | 0.91102334 | -0.13247043 |
| O | -0.58652907 | 4.18147068 | -0.81257591 |
| O | 2.59203816 | 1.25639209 | 0.09505895 |
| O | -0.62352327 | 2.18700225 | 2.97180180 |
| C | -0.51794937 | 1.87568685 | 1.86598424 |
| C | 1.44091545 | 1.28204284 | 0.09760814 |
| C | -0.49501590 | 3.09258423 | -0.44267807 |
| C | 0.08789916 | -2.02503152 | -1.45483739 |
| H | 1.09851479 | -2.42498358 | -1.33572680 |
| H | 0.03270865 | -1.44422535 | -2.37938684 |
| H | -0.62678416 | -2.85125118 | -1.50053478 |
| H | -0.15228133 | -1.38180499 | -0.60419182 |

## TS: $^{3}Fe(CO)_4$ + $CH_4$ -2145.49 (-489.73)

| | | | |
|---|---|---|---|
| Fe | 0.09287166 | -0.24812821 | -0.06899336 |
| O | -2.62942112 | -0.79307697 | -1.28455203 |
| C | -1.91994720 | -0.71776795 | -0.37157109 |
| O | -0.52744035 | 2.51399774 | -0.93888924 |
| O | 3.00176103 | 0.06150096 | -0.40835124 |
| O | 0.11474714 | 0.40050811 | 2.76521106 |
| C | 0.08113471 | 0.10208173 | 1.64192049 |
| C | 1.86912714 | -0.05348660 | -0.23431332 |
| C | -0.34185369 | 1.40834172 | -0.68014349 |
| C | 0.32374573 | -2.18734255 | -0.71232717 |
| H | 1.28589253 | -2.63674876 | -0.45812314 |
| H | 0.28222911 | -1.98112995 | -1.79171821 |
| H | -0.48699516 | -2.86696593 | -0.43884361 |
| H | 0.08642487 | -1.23453928 | 1.11729754 |

## P: $^{3}Fe(CO)_4$ + $CH_4$ -2164.77

| | | | |
|---|---|---|---|
| Fe | 0.53713843 | -0.17975285 | 0.34830057 |
| O | -2.11315200 | 0.15966216 | -0.90648861 |
| C | -1.06587002 | -0.34682487 | -0.64845705 |
| O | -0.67984279 | 2.09690269 | 1.68883136 |
| O | 1.85470168 | 1.37033110 | -1.78468262 |
| O | -0.16380112 | -2.15566814 | 2.43210369 |
| C | 0.10806910 | -1.44473225 | 1.56702666 |
| C | 1.34687047 | 0.70277584 | -0.99430482 |
| C | -0.21052383 | 1.19252145 | 1.15308522 |
| C | 2.11558875 | 0.37391197 | 1.55797932 |
| H | 2.52266296 | 1.36170125 | 1.33335893 |
| H | 2.83618817 | -0.40099241 | 1.26398976 |
| H | 1.89843995 | 0.29313169 | 2.62497843 |
| H | -0.77273924 | -1.32203771 | -1.16249410 |

### RC: $^{3}Fe(CO)_4$ +$CH_3Cl$ -2172.42

| | | | |
|---|---|---|---|
| Fe | -0.34069632 | -1.67506325 | -1.37061419 |
| O | -0.47406155 | -2.87492200 | -4.02375744 |
| C | -0.42144317 | -2.38517732 | -2.98054481 |
| O | -0.63829501 | -4.20028798 | 0.05399342 |
| O | -3.16247606 | -0.80021143 | -1.19110892 |
| O | 2.60771639 | -1.49123154 | -1.19658068 |
| C | 1.45762776 | -1.51070099 | -1.24755242 |
| C | -2.04988181 | -1.09147154 | -1.24570993 |
| C | -0.52031123 | -3.19276841 | -0.49559636 |
| C | -0.60104435 | 2.51967017 | 1.60368051 |
| H | -1.47193556 | 2.82564098 | 2.18763196 |
| H | 0.31527905 | 2.71717560 | 2.16441144 |
| H | -0.66868477 | 1.45759474 | 1.35493272 |
| Cl | -0.56405741 | 3.45515372 | 0.10244891 |

### TS: $^{3}Fe(CO)_4$ +$CH_3Cl$ -2129.10 (-916.88)

| | | | |
|---|---|---|---|
| Fe | -0.05221000 | -0.23784249 | -0.00622311 |
| O | -0.14607620 | -0.44530647 | -2.91364074 |
| C | -0.10720695 | -0.34765992 | -1.76725107 |
| O | -0.46082747 | -3.08660011 | 0.48799131 |
| O | -2.95594626 | 0.30805302 | 0.03917483 |
| O | 2.88446525 | -0.55418660 | -0.09168333 |
| C | 1.75458150 | -0.35642512 | -0.00743569 |
| C | -1.81453089 | 0.17052988 | 0.07233063 |
| C | -0.29767048 | -1.95444520 | 0.33843918 |
| C | 0.18794200 | 1.10724840 | 1.84668837 |
| H | -0.64572735 | 1.66329919 | 2.26519501 |
| H | 1.16427197 | 1.39597302 | 2.22453683 |
| H | 0.03111061 | 0.01852790 | 2.02368846 |
| Cl | 0.34673782 | 2.44391468 | 0.13213788 |

### P: $^{3}Fe(CO)_4$ +$CH_3Cl$ -2142.99

| | | | |
|---|---|---|---|
| Fe | -0.06045095 | -0.33650518 | 0.25899106 |
| O | -0.04300957 | 0.20371117 | -2.45874560 |
| C | 0.04897961 | 0.67541623 | -1.37204356 |
| O | -0.48334251 | -3.27620888 | -0.01057861 |
| O | -2.90223955 | 0.31461856 | 0.54804831 |
| O | 2.85913470 | -0.51481196 | 0.41035723 |
| C | 1.71479310 | -0.43893406 | 0.33025335 |
| C | -1.78805708 | 0.06521306 | 0.41412643 |
| C | -0.32165081 | -2.14311884 | 0.07930987 |
| C | 0.08738189 | 0.35356021 | 2.17316838 |
| H | 0.22580141 | 1.43702679 | 2.08907176 |
| H | 0.94392087 | -0.08725285 | 2.69046601 |
| H | -0.82453783 | 0.14204165 | 2.73836349 |
| Cl | 0.30969799 | 2.45304879 | -1.15536523 |

### RC: $^{3}Fe(CO)_4$ + $C_2H_6$ -2589.38

| | | | |
|---|---|---|---|
| Fe | -0.59072821 | 0.25245306 | 1.45241591 |
| O | -3.36548156 | 0.79878767 | 2.35461986 |
| C | -2.30123265 | 0.58644968 | 1.96058766 |
| O | 0.76496120 | 2.19334485 | 3.16564208 |
| O | 1.63347738 | -0.19525209 | -0.46218390 |
| O | -0.06439036 | -2.02463590 | 3.20853907 |
| C | -0.28158435 | -1.13616559 | 2.49999958 |
| C | 0.73748872 | -0.01310671 | 0.24092336 |
| C | 0.22931159 | 1.43667713 | 2.47420375 |
| C | -2.89789190 | 1.94557280 | -2.18945346 |
| H | -2.14871603 | 2.06650860 | -1.39283177 |
| H | -3.80576939 | 2.47993853 | -1.87159775 |
| H | -2.51222058 | 2.45684560 | -3.08416333 |
| C | -3.18417143 | 0.47822938 | -2.46642581 |
| H | -3.56770668 | -0.03182338 | -1.57021995 |
| H | -2.27703234 | -0.05536940 | -2.78740173 |
| H | -3.93580970 | 0.35637379 | -3.26045371 |

### TS: $^{3}Fe(CO)_4$ + $C_2H_6$ -2512.46 (-1317.7)

| | | | |
|---|---|---|---|
| Fe | -0.04136211 | -0.16090204 | -0.22890819 |
| O | -0.20957964 | -0.87958140 | -3.05326855 |
| C | -0.14121423 | -0.58592087 | -1.93892560 |
| O | -0.41525712 | -2.88147552 | 0.75281234 |
| O | -2.92757937 | 0.40142484 | -0.02945085 |
| O | 2.89178524 | -0.43996362 | -0.15478421 |
| C | 1.75278531 | -0.26193398 | -0.14971060 |
| C | -1.78552452 | 0.25021603 | -0.07309207 |
| C | -0.26652571 | -1.80262801 | 0.37057845 |
| C | 0.17209802 | 0.89948577 | 2.50014276 |
| H | 0.31922761 | 1.85875619 | 2.99379841 |
| H | 1.00608708 | 0.20587278 | 2.58097933 |
| H | -0.81796333 | 0.46537014 | 2.62056380 |
| H | 1.24077310 | 2.22449752 | 0.82079858 |
| C | 0.26646136 | 1.86015994 | 0.49050440 |
| H | -0.55018281 | 2.48299791 | 0.85967168 |
| H | 0.25978308 | 1.98041451 | -0.61757049 |

### P: $^{3}Fe(CO)_4$ + $C_2H_6$ -2538.23

| | | | |
|---|---|---|---|
| Fe | -0.53238367 | -1.81860822 | -1.26600307 |
| O | -0.08612307 | -0.69001082 | -3.90662522 |
| C | -0.26477948 | -1.14495502 | -2.86266356 |
| O | -0.84282299 | -4.59700849 | -2.10279719 |
| O | -3.39359593 | -1.20976549 | -1.12071905 |
| O | 2.29234496 | -2.03467013 | -0.52353996 |
| C | 1.17648430 | -1.97478895 | -0.79851145 |
| C | -2.27438015 | -1.47433683 | -1.16157109 |
| C | -0.72203363 | -3.50135787 | -1.76552450 |
| C | -0.65621501 | -3.93234701 | 2.96495923 |
| H | -1.55006977 | -3.38295202 | 2.69189497 |
| H | 0.16829784 | -4.00900554 | 2.26527585 |
| H | -0.58509244 | -4.40259182 | 3.93921808 |
| H | 0.59632573 | 0.42459440 | -0.34756403 |
| C | -0.38369545 | -0.04345257 | -0.22439488 |
| H | -0.52912288 | -0.30407897 | 0.83106820 |
| H | -1.14966928 | 0.67553990 | -0.52606716 |

### RC: $^{1}Fe(PH_3)_4$ + $CH_3Cl$ -2158.16

| | | | |
|---|---|---|---|
| P | -0.80375100 | 0.45387400 | -2.21997000 |
| P | -2.33868000 | 0.36057100 | 1.08215400 |
| H | -1.45736400 | -0.74994700 | -2.64997500 |
| P | 0.52325200 | 0.12795800 | 0.37936100 |
| H | 1.48977600 | -0.45742100 | -0.49854700 |
| H | 0.47876100 | 0.01358900 | -2.68250600 |
| H | -3.09739600 | 3.63341600 | -0.73791200 |
| P | -2.80448700 | 2.28037800 | -1.10973300 |
| H | -2.98792700 | 2.50718800 | -2.51091900 |
| H | -4.13203500 | 1.80343500 | -0.89183200 |
| H | -1.12315300 | 1.14652800 | -3.43358700 |
| H | -2.64327800 | 0.86805700 | 2.39866200 |
| H | -3.71698600 | 0.08321600 | 0.81272000 |
| H | -2.05856100 | -0.94730800 | 1.59096000 |
| H | 1.49968900 | 0.69998200 | 1.25915300 |
| H | 0.33534100 | -1.05484100 | 1.15623700 |
| C | -0.23667100 | 4.01191400 | 1.63766700 |
| Cl | -0.01087700 | 3.22878900 | 0.02500500 |
| H | 0.33816000 | 4.94131700 | 1.63448900 |
| H | 0.13060300 | 3.32545200 | 2.40248900 |
| H | -1.30151100 | 4.20923600 | 1.77282500 |

### TS: $^{1}Fe(PH_3)_4$ + $CH_3Cl$ -2132.67 (-453.36)

| | | | |
|---|---|---|---|
| Fe | -0.19999100 | 0.51819900 | 0.31491100 |
| P | -1.54918900 | -0.97429200 | 0.80668200 |
| P | 0.92658500 | -0.09182600 | -1.34398700 |
| H | -2.56311800 | -1.43201700 | -0.09313200 |
| P | -1.61505800 | 1.40879700 | -0.99317700 |
| H | -2.25908200 | 0.61996000 | -1.98667700 |
| H | -2.47096900 | -0.85709200 | 1.91266800 |
| H | 1.13422000 | -0.35999900 | 3.03087600 |
| P | 1.25830600 | -0.33379300 | 1.60101200 |
| H | 1.59463200 | -1.71075400 | 1.47905200 |
| H | 2.59813100 | 0.15744500 | 1.61582400 |
| H | -1.14473700 | -2.29479300 | 1.18083000 |
| H | 1.96649200 | 0.68080400 | -1.97546300 |
| H | 1.72497800 | -1.27958700 | -1.32648600 |
| H | 0.31841200 | -0.42291500 | -2.59647300 |
| H | -2.81738500 | 2.04949600 | -0.54093600 |
| H | -1.23893700 | 2.47951700 | -1.85871500 |
| Cl | 0.50056100 | 2.69078100 | 0.99338400 |
| C | -1.02166600 | 2.09593000 | 2.28056000 |
| H | -0.76272800 | 1.25000800 | 2.91186800 |
| H | -1.99187100 | 1.99200600 | 1.80198900 |
| H | -0.94022500 | 3.03799800 | 2.82003400 |

### P: $^{1}Fe(PH_3)_4$ + $CH_3Cl$ -2191.42

| | | | |
|---|---|---|---|
| Fe | -0.59157902 | 0.25582855 | 0.45813640 |
| P | 0.83842754 | -1.14415962 | -0.09902335 |
| P | -2.30397809 | -0.80935438 | -0.23311807 |
| Cl | -2.11569916 | 1.88516911 | 1.11776584 |
| P | -0.75972736 | -0.36472944 | 2.48672131 |
| H | 0.09321656 | -1.36232815 | 3.04549222 |
| H | 1.69637477 | -1.73062237 | 0.87726443 |
| H | -0.12881374 | 2.85991644 | -1.05606240 |
| P | -0.40424292 | 1.48676814 | -1.26733223 |
| H | 0.60079006 | 1.25882890 | -2.25383865 |
| H | -1.48224116 | 1.65903498 | -2.17877592 |
| H | 1.61436710 | 0.86413283 | 1.93993007 |
| C | 1.02692677 | 1.35403347 | 1.14924363 |
| H | 0.60605542 | 2.27608831 | 1.56564178 |
| H | 1.75813305 | 1.63593006 | 0.37709519 |
| H | -0.55032894 | 0.61820700 | 3.48464782 |
| H | -1.97577205 | -0.88951974 | 3.00428730 |
| H | 0.51278586 | -2.37348579 | -0.75005351 |
| H | 1.88528162 | -0.78715939 | -0.99899407 |
| H | -3.17038514 | -0.24073174 | -1.20938267 |
| H | -2.20403945 | -2.08625409 | -0.86490849 |
| H | -3.34520472 | -1.18160906 | 0.66387136 |

**RC: $^{1}Fe(PH_3)_4CH_3Cl + MgClCH_3$** -2769.08

| | | | |
|---|---|---|---|
| Fe | 0.22311800 | -2.16102900 | -0.87487900 |
| P | 1.46079000 | -3.82768700 | -0.90393800 |
| P | -1.24953700 | -2.91655300 | -2.23418200 |
| Cl | -1.08636300 | -0.17112800 | -0.82471400 |
| P | -0.79084200 | -2.81495600 | 0.90379600 |
| H | -0.17840100 | -3.78750800 | 1.73872800 |
| H | 1.79162100 | -4.48957800 | 0.31188200 |
| H | 1.79676600 | 0.20212000 | -2.02970700 |
| P | 1.36255100 | -1.10860400 | -2.34846900 |
| H | 2.62032400 | -1.60803800 | -2.78824700 |
| H | 0.84567200 | -0.81373200 | -3.64039400 |
| H | 1.71068900 | -1.96127600 | 1.40117200 |
| C | 1.62588900 | -1.41343600 | 0.45186000 |
| H | 1.30835400 | -0.39389500 | 0.69418300 |
| H | 2.65107000 | -1.35275900 | 0.05753800 |
| H | -1.06486200 | -1.83424400 | 1.88933800 |
| H | -2.07821800 | -3.41963900 | 0.86580300 |
| H | 1.13999200 | -5.00927000 | -1.63761900 |
| H | 2.79880400 | -3.71637600 | -1.37898800 |
| H | -1.51652800 | -2.27215100 | -3.47539300 |
| H | -1.14429500 | -4.22970000 | -2.77859100 |
| H | -2.61462000 | -3.03447800 | -1.84802500 |
| H | -2.25047700 | 4.19131300 | 0.71899300 |
| Mg | -1.71611600 | 1.54332000 | 0.91665100 |
| Cl | -1.86202500 | 0.56030300 | 2.95480700 |
| C | -1.97663900 | 3.41098600 | -0.00547000 |
| H | -1.06601500 | 3.75723000 | -0.51600100 |
| H | -2.76879600 | 3.39512400 | -0.76809100 |

**TS: $^{1}Fe(PH_3)_4CH_3Cl + MgClCH_3$** -2747.17 (-76.54)

| | | | |
|---|---|---|---|
| Fe | 0.64818700 | -2.15582100 | -0.93225400 |
| P | 1.66597700 | -3.67093800 | -1.87797000 |
| P | -1.17887300 | -2.24085200 | -2.09376800 |
| Cl | -2.30671500 | -1.23342100 | 0.93769500 |
| P | -0.10920800 | -3.47677000 | 0.61018100 |
| H | 0.64486400 | -4.66679100 | 0.81372600 |
| H | 1.80255500 | -4.94820000 | -1.26516600 |
| H | 2.51287800 | 0.20967400 | -1.78059800 |
| P | 1.54273100 | -0.70074000 | -2.26970200 |
| H | 2.32199800 | -1.19807300 | -3.35498700 |
| H | 0.78305400 | 0.21188300 | -3.04409000 |
| H | 2.45483800 | -2.73592500 | 0.99918400 |
| C | 2.22251800 | -1.92195600 | 0.29887600 |
| H | 1.88192700 | -1.05959000 | 0.89493000 |
| H | 3.17239400 | -1.63749700 | -0.17280100 |
| H | -0.10104600 | -3.02333700 | 1.94812600 |
| H | -1.39317100 | -4.06981100 | 0.57513000 |
| H | 1.27171100 | -4.14515400 | -3.16421900 |
| H | 3.04220900 | -3.49112400 | -2.19130600 |
| H | -1.93931400 | -1.07281100 | -2.35697500 |
| H | -1.08887500 | -2.66779400 | -3.45167600 |
| H | -2.26715900 | -3.07791500 | -1.73946200 |
| H | 0.70642300 | 1.81567000 | 0.45904300 |
| Mg | -1.94084900 | 1.11175700 | 1.11875400 |
| Cl | -3.32675300 | 2.44224400 | 2.28971800 |
| C | -0.18598200 | 1.44055500 | -0.06078000 |
| H | 0.02615300 | 0.40163000 | -0.38796500 |
| H | -0.31758700 | 2.05758200 | -0.96093300 |

**P: $^{1}Fe(PH_3)_4CH_3Cl + MgClCH_3$** -2779.67

| | | | |
|---|---|---|---|
| Fe | 0.14317700 | 0.33831600 | 0.36281800 |
| P | 0.20185000 | -1.76178400 | 0.49668600 |
| P | 2.24617600 | 0.58436500 | 0.27804800 |
| Cl | -0.53977300 | 5.64742100 | 2.16217200 |
| P | -0.21781900 | 0.35309800 | -1.72378800 |
| H | -0.64669200 | -0.82388500 | -2.39906300 |
| H | -0.39733100 | -2.57586600 | -0.50930200 |
| H | -1.04886700 | 1.53221000 | 2.91771300 |
| P | -0.07012200 | 0.61523000 | 2.45191300 |
| H | -0.45183300 | -0.46622800 | 3.29451200 |
| H | 0.98753400 | 1.08779600 | 3.28297300 |
| H | -2.34994300 | -0.50782100 | -0.36391200 |
| C | -1.92819300 | 0.09806200 | 0.45250300 |
| H | -2.42113000 | 1.07730300 | 0.40304500 |
| H | -2.28742400 | -0.38862700 | 1.37204000 |
| H | -1.23419400 | 1.20479800 | -2.23088900 |
| H | 0.77714600 | 0.72134300 | -2.67609000 |
| H | 1.42182900 | -2.50066200 | 0.55652500 |
| H | -0.41647500 | -2.43751100 | 1.58970400 |
| H | 2.93238500 | 1.42165100 | 1.20825000 |
| H | 3.14954300 | -0.51195500 | 0.40962000 |
| H | 2.88910000 | 1.13682200 | -0.87037400 |
| C | 0.00242200 | 2.43113000 | 0.23343500 |
| H | 0.50734700 | 2.91424300 | 1.08552200 |
| H | -1.08146300 | 2.63687900 | 0.26609500 |
| H | 0.43516700 | 2.80124300 | -0.70962900 |
| Mg | -0.41049000 | 4.84484300 | 0.09438300 |
| Cl | -0.43457200 | 5.41420800 | -2.05400200 |

**RC: $^{1}Fe(PH_3)_4 + C_2H_6$** -2569.22

| | | | |
|---|---|---|---|
| Fe | -0.22673700 | 0.30274100 | 0.28184800 |
| P | -1.33735100 | -1.44900000 | 0.63656700 |
| P | 0.87892500 | -0.46395000 | -1.33607000 |
| C | 0.85196400 | 2.06306700 | -0.02781000 |
| P | -1.67780600 | 1.31826800 | -0.84341100 |
| H | -3.03574300 | 0.88401900 | -0.91144400 |
| H | -2.76401000 | -1.45320100 | 0.56068800 |
| H | 1.53139800 | 1.07106300 | 2.64424000 |
| P | 1.22669600 | -0.00301200 | 1.76444200 |
| H | 1.08318000 | -0.98921200 | 2.78605500 |
| H | 2.58254300 | -0.32213100 | 1.45292300 |
| H | -2.38582700 | 1.13237700 | 1.73313000 |
| C | -1.29517800 | 1.11099100 | 1.88287700 |
| H | -0.97302000 | 2.14696100 | 2.04755500 |
| H | -1.15643000 | 0.57511600 | 2.83469500 |
| H | -1.97524300 | 2.66642100 | -0.50507200 |
| H | -1.53562200 | 1.54995300 | -2.24459500 |
| H | -1.17681300 | -2.64781200 | -0.12482300 |
| H | -1.29533200 | -2.10902100 | 1.90298200 |
| H | 2.30628700 | -0.51450400 | -1.30059600 |
| H | 0.72757800 | -1.79678000 | -1.82943100 |
| H | 0.82564700 | 0.14511500 | -2.62730500 |
| H | 1.94211100 | 1.94918200 | 0.07718100 |
| H | 0.53544900 | 2.81549100 | 0.70551700 |
| H | 0.71455600 | 2.50774000 | -1.02570900 |

**TS: $^{1}Fe(PH_3)_4 + C_2H_6$** -2522.4 (-675.54)

| | | | |
|---|---|---|---|
| Fe | -0.31137500 | 0.43737000 | 0.27140400 |
| P | -1.52387700 | -1.03885900 | 1.11043800 |
| P | 0.80802100 | -0.50465800 | -1.22067400 |
| H | -1.22659600 | 3.27307700 | 1.52112500 |
| P | -1.78024600 | 0.87070100 | -1.19244500 |
| H | -2.22609800 | -0.14806000 | -2.07905800 |
| H | -1.00721400 | -2.18636600 | 1.79346400 |
| H | 1.16292100 | 0.36960100 | 3.02145600 |
| P | 1.23179000 | -0.02969600 | 1.64718800 |
| H | 1.57169300 | -1.38669500 | 1.90422500 |
| H | 2.58581400 | 0.41597600 | 1.47911900 |
| H | -1.99034000 | 1.72518900 | 1.42536400 |
| C | 0.35265200 | 2.58839500 | 0.32412800 |
| H | 1.11183700 | 2.08693000 | -0.28759200 |
| C | -1.00860100 | 2.19964000 | 1.52584900 |
| H | -3.09157400 | 1.33978600 | -0.84266600 |
| H | -1.58649500 | 1.86184200 | -2.20790300 |
| H | -2.51494300 | -0.78285000 | 2.13060900 |
| H | -2.43644400 | -1.81848600 | 0.32978700 |
| H | 0.20687400 | -1.13574100 | -2.35663400 |
| H | 1.82657700 | 0.13307800 | -2.02734800 |
| H | 1.65248400 | -1.62612200 | -0.93937900 |
| H | 0.90749200 | 3.13153500 | 1.09507500 |
| H | -0.59327700 | 1.96585700 | 2.50782500 |
| H | -0.15468700 | 3.31234700 | -0.31572700 |

**P: $^{1}Fe(PH_3)_4$** -2779.67

| | | | |
|---|---|---|---|
| Fe | -0.14723100 | 0.33717600 | 0.27365000 |
| P | -1.62410600 | -0.92102100 | 0.98472200 |
| P | 1.09114200 | 0.13744700 | -1.37373300 |
| H | -1.85757900 | -2.15943800 | 0.29884100 |
| P | -1.55659700 | 1.49536800 | -0.78076900 |
| H | -2.97372100 | 1.33644600 | -0.66771600 |
| H | -3.01840300 | -0.58808600 | 1.04442100 |
| H | 1.92169900 | 0.46503800 | 2.43841000 |
| P | 1.27258900 | -0.49179200 | 1.59097100 |
| H | 0.95811300 | -1.41593900 | 2.63684700 |
| H | 2.47293000 | -1.16044800 | 1.19873600 |
| H | -1.61640900 | -1.54152600 | 2.27921200 |
| H | 2.10476800 | 1.07171900 | -1.80596700 |
| H | 1.97717400 | -0.97670900 | -1.49676300 |
| H | 0.54845500 | 0.02133400 | -2.69019000 |
| H | -1.54253900 | 2.89053900 | -0.45185900 |
| H | -1.57153100 | 1.69212800 | -2.19621600 |

**RC: $Fe^{-II}(CO)_4 + CH_4$** -2215.96

| | | | |
|---|---|---|---|
| Fe | 0.04042068 | -0.07953660 | 0.37040726 |
| O | 2.37030492 | -1.33120920 | -0.88240770 |
| C | 1.41436381 | -0.81835428 | -0.36870604 |
| O | 0.13855524 | -0.56349707 | 3.25349800 |
| O | -2.43317279 | -1.22958468 | -0.69200069 |
| O | 0.09008608 | 2.81045283 | -0.08364265 |
| C | 0.06926760 | 1.62398981 | 0.09514918 |
| C | -1.41770164 | -0.75702815 | -0.26092077 |
| C | 0.09695582 | -0.36713753 | 2.07044411 |
| C | -0.33665626 | 0.65816461 | -4.41288564 |
| H | 0.22743267 | -0.11469563 | -4.94613322 |
| H | 0.08059650 | 1.64353315 | -4.64780814 |
| H | -1.38655628 | 0.62349983 | -4.72112994 |
| H | -0.26569813 | 0.47793505 | -3.33480499 |

**TS: $Fe^{-II}(CO)_4 + CH_4$** -2139.72 (-706.37)

| | | | |
|---|---|---|---|
| Fe | -0.41896107 | -0.05366525 | -0.27208325 |
| O | 3.42770515 | 0.00534708 | 0.15242348 |
| C | 2.69430736 | -0.20745744 | -0.72903278 |
| O | 0.33264914 | -1.85234246 | 1.91943673 |
| O | -3.28757926 | 0.24640986 | 0.09796473 |
| O | 0.58058624 | 2.61177397 | 0.34381717 |
| C | 0.22616011 | 1.49964068 | 0.07392336 |
| C | -2.11519753 | 0.09061470 | -0.12596591 |
| C | 0.05394322 | -1.14074240 | 0.99646090 |
| C | -0.14353484 | -1.21236011 | -2.06707934 |
| H | -0.39535932 | -2.20503997 | -1.66542393 |
| H | 0.89784314 | -1.24492364 | -2.42567600 |
| H | -0.79969914 | -1.05527350 | -2.93940496 |
| H | -0.22820453 | 0.26771951 | -1.74033853 |

**P: $Fe^{-II}(CO)_4 + CH_4$** -2147.21

| | | | |
|---|---|---|---|
| Fe | -0.41460779 | -0.00117577 | -0.30304795 |
| O | 3.58658255 | -0.08127604 | 0.14206203 |
| C | 2.88534152 | -0.48661978 | -0.68956496 |
| O | 0.40068555 | -1.76910153 | 1.85472799 |
| O | -3.28854077 | 0.09075564 | 0.07422721 |
| O | 0.41252570 | 2.69910704 | 0.44248609 |
| C | 0.09480343 | 1.57586001 | 0.12757707 |
| C | -2.11144084 | 0.05658435 | -0.15991349 |
| C | 0.14118800 | -1.07661157 | 0.91101207 |
| C | -0.37216638 | -1.25934774 | -1.95872124 |
| H | -0.89489995 | -2.21217186 | -1.76379176 |
| H | 0.66751865 | -1.50444408 | -2.23595467 |
| H | -0.85618324 | -0.80694594 | -2.84358237 |
| H | 0.32591873 | 1.12830829 | -1.18410179 |

### RC($S_N2$): $Fe^{-II}(CO)_4 + CH_4$ -2215.98

| | | | |
|---|---|---|---|
| Fe | 0.02909300 | -0.06980300 | 0.35290900 |
| O | 2.32309900 | -1.35374200 | -0.93980700 |
| C | 1.38269900 | -0.82254200 | -0.41766900 |
| O | 0.18714800 | -0.57491400 | 3.22978300 |
| O | -2.47324900 | -1.24676800 | -0.61124800 |
| O | 0.05095800 | 2.82676300 | -0.08719000 |
| C | 0.04359100 | 1.63947900 | 0.08428300 |
| C | -1.44653100 | -0.75999200 | -0.22666500 |
| C | 0.12567700 | -0.36537800 | 2.05061400 |
| C | -0.17640800 | 0.65121900 | -4.31380500 |
| H | 0.63462500 | 0.09617200 | -4.79661500 |
| H | -0.05976500 | 1.72032500 | -4.52007200 |
| H | -1.13867800 | 0.30685500 | -4.70701000 |
| H | -0.14070000 | 0.48184500 | -3.23213800 |

### TS($S_N2$): $Fe^{-II}(CO)_4 + CH_4$ -2150.98 (-577.92)

| | | | |
|---|---|---|---|
| Fe | -0.11798500 | 0.23002300 | -1.62465600 |
| O | 2.33858800 | -1.18957500 | -2.33510500 |
| C | 1.33617300 | -0.60342200 | -2.09661100 |
| O | -0.00193300 | -0.25201200 | 1.23061200 |
| O | -2.68210100 | -1.08888900 | -2.11533400 |
| O | -0.04819100 | 3.15151200 | -1.50321700 |
| C | -0.07875700 | 1.97068600 | -1.60337800 |
| C | -1.64048100 | -0.54333000 | -1.96503300 |
| C | -0.04923000 | -0.05470900 | 0.06122000 |
| C | -0.23482000 | 0.65691000 | -4.15714600 |
| H | -0.27702600 | -0.36288900 | -4.50929900 |
| H | -0.33532300 | 1.01859700 | -6.29818600 |
| H | -1.14660700 | 1.23366200 | -4.19953900 |
| H | 0.69481100 | 1.18929100 | -4.29175300 |

### P($S_N2$): $Fe^{-II}(CO)_4 + CH_4$ -2157.9

| | | | |
|---|---|---|---|
| Fe | -0.09130800 | 0.21024500 | -1.48933300 |
| O | 2.37809100 | -1.24140700 | -2.05085900 |
| C | 1.37981100 | -0.64931000 | -1.85161800 |
| O | -0.08033900 | -0.30786900 | 1.36415800 |
| O | -2.64939000 | -1.09845200 | -2.00357000 |
| O | -0.01034900 | 3.11759800 | -1.24171000 |
| C | -0.04260100 | 1.94733600 | -1.36978800 |
| C | -1.61535000 | -0.56417400 | -1.82394000 |
| C | -0.08474500 | -0.09768400 | 0.20250300 |
| C | -0.09790900 | 0.61015800 | -3.66150600 |
| H | -0.12987000 | -0.33627100 | -4.19897200 |
| H | -0.10953200 | 1.15583900 | -6.61153300 |
| H | -0.97371600 | 1.20602700 | -3.91348200 |
| H | 0.80653300 | 1.15450600 | -3.92841000 |

### RC($S_N2$): $Fe^{-II}(CO)_4 + CH_3Cl$ -2188.28

| | | | |
|---|---|---|---|
| Fe | -0.10969400 | 0.17719700 | -1.30864600 |
| O | 2.22571900 | -1.17021900 | -2.45511400 |
| C | 1.26653100 | -0.61002800 | -2.00732800 |
| O | 0.03659900 | -0.29212400 | 1.56681000 |
| O | -2.63427000 | -1.03935200 | -2.16077200 |
| O | -0.06591800 | 3.08695500 | -1.65187400 |
| C | -0.08323100 | 1.89519000 | -1.53420900 |
| C | -1.59873400 | -0.53325400 | -1.83586500 |
| C | -0.02137000 | -0.09789800 | 0.38818200 |
| C | -0.26217100 | 0.82895500 | -5.16028800 |
| H | 0.63236600 | 0.24330400 | -4.95901900 |
| Cl | -0.33268000 | 1.15013000 | -6.97347700 |
| H | -1.15882100 | 0.27864900 | -4.88260200 |
| H | -0.22178000 | 1.78957400 | -4.65113200 |

### TS($S_N2$): $Fe^{-II}(CO)_4 + CH_3Cl$ -2186.46 (-247.24)

| | | | |
|---|---|---|---|
| Fe | -0.10841300 | 0.20201700 | -1.46635300 |
| O | 2.32388200 | -1.19373500 | -2.30689500 |
| C | 1.32744600 | -0.61399300 | -2.01292400 |
| O | -0.00286700 | -0.27188800 | 1.39307300 |
| O | -2.66006300 | -1.09012800 | -2.09019100 |
| O | -0.04965700 | 3.12899400 | -1.49929500 |
| C | -0.07508200 | 1.94009800 | -1.53512400 |
| C | -1.61857700 | -0.55308400 | -1.88472300 |
| C | -0.04492700 | -0.07723700 | 0.22003800 |
| C | -0.25101000 | 0.71934100 | -4.49599000 |
| H | -0.26930300 | -0.36075700 | -4.50258100 |
| Cl | -0.35363700 | 1.08526300 | -6.65014700 |
| H | -1.15192300 | 1.23138900 | -4.19075800 |
| H | 0.69238800 | 1.20068400 | -4.28277000 |

### P($S_N2$): $Fe^{-II}(CO)_4 + CH_3Cl$ -2205.75

| | | | |
|---|---|---|---|
| Fe | 0.03474500 | -0.60306800 | 1.53756300 |
| O | 2.49520900 | -2.05818400 | 0.95524400 |
| C | 1.50062100 | -1.46711400 | 1.17244900 |
| O | 0.05594300 | -1.07007600 | 4.40121000 |
| O | -2.52250200 | -1.90790900 | 1.01977200 |
| O | 0.12171200 | 2.30868300 | 1.69197000 |
| C | 0.08696000 | 1.13472600 | 1.61184400 |
| C | -1.48903200 | -1.37764100 | 1.21095800 |
| C | 0.04764600 | -0.88086600 | 3.23627800 |
| C | 0.01639200 | -0.24133800 | -0.63738900 |
| Cl | -0.01108200 | 0.24866100 | -3.59718200 |
| H | 0.92247800 | 0.29463700 | -0.90338000 |
| H | -0.86107800 | 0.35066100 | -0.87941400 |
| H | -0.02061400 | -1.20127300 | -1.14352800 |

### RC($S_N2$): $Fe^{-II}(CO)_4 + C_2H_6$ -2596.31

| | | | |
|---|---|---|---|
| Fe | -0.01239400 | -0.06893600 | 0.32298200 |
| O | 2.24975400 | -1.22773800 | -1.13183500 |
| C | 1.32107200 | -0.75388200 | -0.53793600 |
| O | 0.37451700 | -0.49982400 | 3.19152100 |
| O | -2.51044600 | -1.37169900 | -0.47805200 |
| O | -0.14384800 | 2.81320800 | -0.17848400 |
| C | -0.09261700 | 1.63138500 | 0.02303200 |
| C | -1.48699900 | -0.83526000 | -0.15627200 |
| C | 0.21683700 | -0.32436900 | 2.01589400 |
| C | -0.33065100 | 0.81213300 | -4.25873200 |
| H | -0.18928700 | -0.05446700 | -4.91906000 |
| C | 0.81691600 | 1.80056000 | -4.38266400 |
| H | -1.29101100 | 1.27669800 | -4.52044500 |
| H | -0.40864300 | 0.44286900 | -3.22945200 |
| H | 1.76655100 | 1.32675400 | -4.10786900 |
| H | 0.67683300 | 2.64256700 | -3.69557400 |
| H | 0.91853300 | 2.20194500 | -5.40272800 |

### TS($S_N2$): $Fe^{-II}(CO)_4 + C_2H_6$ -2532.42 (-313.36)

| | | | |
|---|---|---|---|
| Fe | -0.11344800 | 0.21830000 | -1.56415800 |
| O | 2.35169200 | -1.18082300 | -2.28601800 |
| C | 1.34582000 | -0.60380400 | -2.03837100 |
| O | -0.00605100 | -0.25308000 | 1.29388100 |
| O | -2.66419500 | -1.11494500 | -2.08518600 |
| O | -0.06230600 | 3.14148900 | -1.47723900 |
| C | -0.08527800 | 1.95875600 | -1.55812400 |
| C | -1.62751300 | -0.56445400 | -1.91762900 |
| C | -0.04950900 | -0.06050600 | 0.12351600 |
| C | -0.23147400 | 0.64408000 | -4.10165600 |
| H | -0.26694400 | -0.37742500 | -4.44884100 |
| C | -0.37190700 | 1.09111800 | -6.74210800 |
| H | -1.14691900 | 1.21465100 | -4.14037800 |
| H | 0.69552500 | 1.18161400 | -4.23247400 |
| H | 0.48448100 | 0.60346000 | -7.23889000 |
| H | -0.34412800 | 2.18046900 | -6.91688900 |
| H | -1.32041800 | 0.66944200 | -7.11693000 |

### P($S_N2$): $Fe^{-II}(CO)_4 + C_2H_6$ -2534.49

| | | | |
|---|---|---|---|
| Fe | -0.11396900 | 0.21695500 | -1.54842000 |
| O | 2.35939600 | -1.19727000 | -2.19629500 |
| C | 1.35497000 | -0.61810700 | -1.97144000 |
| O | 0.00102100 | -0.25943200 | 1.30928800 |
| O | -2.66759200 | -1.13349600 | -1.98639700 |
| O | -0.06186300 | 3.13290300 | -1.37060300 |
| C | -0.08475600 | 1.95718600 | -1.48004100 |
| C | -1.63431000 | -0.57964600 | -1.84445900 |
| C | -0.04551800 | -0.06565300 | 0.14307200 |
| C | -0.21766200 | 0.59830600 | -3.82175300 |
| H | -0.25525400 | -0.37205500 | -4.30792500 |
| C | -0.39120700 | 1.08870500 | -6.77640000 |
| H | -1.11743100 | 1.17652100 | -4.00964700 |
| H | 0.67768100 | 1.14923700 | -4.09451800 |
| H | 0.44117700 | 0.61776700 | -7.34144000 |
| H | -0.38692900 | 2.17502600 | -7.00803100 |
| H | -1.34350300 | 0.66430700 | -7.15874400 |

### $CH_4$ -554.09

| | | | |
|---|---|---|---|
| C | 0.00000000 | 0.00000000 | 0.00000000 |
| H | 0.63276400 | -0.63276400 | 0.63276400 |
| H | -0.63276400 | -0.63276400 | -0.63276400 |
| H | -0.63276400 | 0.63276400 | 0.63276400 |
| H | 0.63276400 | 0.63276400 | -0.63276400 |

### $CH_3Cl$ -516.57

| | | | |
|---|---|---|---|
| C | 0.00000000 | 0.00000000 | -2.00508000 |
| Cl | 0.00000000 | 0.00000000 | -0.23687800 |
| H | -0.51699900 | -0.89546800 | -2.35691600 |
| H | -0.51699900 | 0.89546800 | -2.35691600 |
| H | 1.03399700 | 0.00000000 | -2.35691600 |

### $C_2H_6$ -933.49

| | | | |
|---|---|---|---|
| C | 0.00000000 | 0.00000000 | -0.76004500 |
| C | 0.00000000 | 0.00000000 | 0.76004500 |
| H | -0.00001600 | -1.02019200 | -1.16239400 |
| H | -0.88350400 | 0.51011000 | -1.16239400 |
| H | 0.88352000 | 0.51008200 | -1.16239400 |
| H | 0.88352000 | -0.51008200 | 1.16239400 |
| H | -0.88350400 | -0.51011000 | 1.16239400 |
| H | -0.00001600 | 1.02019200 | 1.16239400 |

### $Pd(CO)_2$ -775.51

| | | | |
|---|---|---|---|
| Pd | 0.00000000 | 0.00000000 | -0.99564909 |
| C | 1.81458968 | 0.00000000 | -0.41703934 |
| C | -1.81458968 | 0.00000000 | -0.41703934 |
| O | 2.84077579 | 0.00000000 | 0.09746426 |
| O | -2.84077579 | 0.00000000 | 0.09746426 |

### $Pd(PH_3)_2$ -785.19

| | | | |
|---|---|---|---|
| Pd | 0.00000000 | 0.00000000 | 0.00000000 |
| H | 0.66596013 | 1.01127603 | 2.98138684 |
| H | 0.66596013 | -1.01127603 | -2.98138684 |
| P | 0.00000000 | 0.00000000 | -2.23097113 |
| H | -1.20877080 | -0.07110037 | -2.98138684 |
| H | 0.54281067 | 1.08237640 | -2.98138684 |
| P | 0.00000000 | 0.00000000 | 2.23097113 |
| H | -1.20877080 | 0.07110037 | 2.98138684 |
| H | 0.54281067 | -1.08237640 | 2.98138684 |

### $^{1}Fe(CO)_4$ -1655.71

| | | | |
|---|---|---|---|
| Fe | 0.00000000 | 0.00000000 | 0.00266480 |
| O | -2.92700731 | 0.00000000 | 0.08271546 |
| C | -1.77694389 | 0.00000000 | 0.02975038 |
| O | 0.00000000 | 2.48259819 | 1.46467953 |
| O | 2.92700731 | 0.00000000 | 0.08271546 |
| O | 0.00000000 | -2.48259819 | 1.46467953 |
| C | 0.00000000 | -1.54757822 | 0.78082679 |
| C | 1.77694389 | 0.00000000 | 0.02975038 |
| C | 0.00000000 | 1.54757822 | 0.78082679 |

## $^{3}Fe(CO)_4$ -1655.73

| | | | |
|---|---|---|---|
| Fe | 0.00000000 | 0.00000000 | 0.03357252 |
| O | -2.90411507 | 0.00000000 | -0.53617704 |
| C | -1.76533556 | 0.00000000 | -0.36612355 |
| O | 0.00000000 | 2.14900907 | 2.00272278 |
| O | 2.90411507 | 0.00000000 | -0.53617704 |
| O | 0.00000000 | -2.14900907 | 2.00272278 |
| C | 0.00000000 | -1.30861404 | 1.21243264 |
| C | 1.76533556 | 0.00000000 | -0.36612355 |
| C | 0.00000000 | 1.30861404 | 1.21243264 |

## $Fe^{-II}(CO)_4$ -1659.49

| | | | |
|---|---|---|---|
| Fe | 0.00000000 | 0.00000000 | 0.41153541 |
| O | -2.38251414 | 0.00000000 | -1.28917807 |
| C | -1.40641172 | 0.00000000 | -0.59007386 |
| O | 0.00000000 | 2.38249942 | 2.11227250 |
| O | 2.38251414 | 0.00000000 | -1.28917807 |
| O | 0.00000000 | -2.38249942 | 2.11227250 |
| C | 0.00000000 | -1.40640662 | 1.41315507 |
| C | 1.40641172 | 0.00000000 | -0.59007386 |
| C | 0.00000000 | 1.40640662 | 1.41315507 |

## $Fe(PH_3)_4$ -1636.72

| | | | |
|---|---|---|---|
| Fe | -0.14723100 | 0.33717600 | 0.27365000 |
| P | -1.62410600 | -0.92102100 | 0.98472200 |
| P | 1.09114200 | 0.13744700 | -1.37373300 |
| H | -1.85757900 | -2.15943800 | 0.29884100 |
| P | -1.55659700 | 1.49536800 | -0.78076900 |
| H | -2.97372100 | 1.33644600 | -0.66771600 |
| H | -3.01840300 | -0.58808600 | 1.04442100 |
| H | 1.92169900 | 0.46503800 | 2.43841000 |
| P | 1.27258900 | -0.49179200 | 1.59097100 |
| H | 0.95811300 | -1.41593900 | 2.63684700 |
| H | 2.47293000 | -1.16044800 | 1.19873600 |
| H | -1.61640900 | -1.54152600 | 2.27921200 |
| H | 2.10476800 | 1.07171900 | -1.80596700 |
| H | 1.97717400 | -0.97670900 | -1.49676300 |
| H | 0.54845500 | 0.02133400 | -2.69019000 |
| H | -1.54253900 | 2.89053900 | -0.45185900 |
| H | -1.57153100 | 1.69212800 | -2.19621600 |

## $MgClCH_3$ -566.84

| | | | |
|---|---|---|---|
| C | 0.00000000 | 0.00000000 | 1.59904100 |
| H | 0.50919900 | 0.88195900 | 1.18772500 |
| H | -1.01839800 | 0.00000000 | 1.18772500 |
| H | 0.50919900 | -0.88195900 | 1.18772500 |
| Mg | 0.00000000 | 0.00000000 | 3.67342100 |
| Cl | 0.00000000 | 0.00000000 | 5.87635300 |

## $MgCl_2$ -201.89

| | | | |
|---|---|---|---|
| Cl | -2.17781600 | 0.00000000 | -5.36323700 |
| Mg | 0.00000000 | 0.00000000 | -5.35707500 |
| Cl | 2.17781600 | 0.00000000 | -5.36323700 |
